# Cell surface polysaccharides in the gut microbiota: occurrence, structure and role

**DOI:** 10.1080/19490976.2025.2536082

**Published:** 2025-09-02

**Authors:** Victor Laplanche, Immacolata Speciale, Cristina De Castro, Nathalie Juge

**Affiliations:** a The Food, Microbiome and Health Institute Strategic Programme, Quadram Institute Bioscience, Norwich Research Park, Norwich, UK; b Department of Chemical Sciences, University of Napoli Federico II, Napoli, Italy

**Keywords:** Gut microbiota, gut commensal bacteria, lactic acid bacteria, cell wall polysaccharides, exopolysaccharides, capsular polysaccharides, microbe-host interactions

## Abstract

The gastrointestinal (GI) tract is colonized by trillions of microorganisms living in a symbiotic relationship with the host. Commensal bacteria in the gut engage in cross-talks with epithelial and immune cells through effector molecules secreted or attached to the cell surface. Although cell surface polysaccharides have mainly been studied in the context of pathogen-host interactions, these are increasingly being recognized as important factors of the symbiotic interaction between the gut microbiota and the host conferring biological activities and physiological functions. In this review, we focus on the structure and role of polysaccharides surrounding the bacterial cell wall, namely capsular polysaccharide (CPS) and cell wall polysaccharides (CWPS), both tightly linked to the cell surface, and exopolysaccharides (EPS) which are loosely attached to the extracellular surface or secreted into the environment. We will focus on structurally characterized CPS, CWPS and EPS from both gut commensal bacteria and food-derived bacteria found in the gut. These polysaccharides show high structural diversity and are important for the bacteria to adapt to the GI environment and/or influence host immune response. The combined diversity of microbes in the gut provides a vast array of glycans that could be harnessed to benefit human health.

## 1. Introduction

The human gut microbiota is a complex, dynamic, and spatially heterogeneous microbial ecosystem whose composition differs across life and in response to environmental factors.^1,^^2,3^ *Firmicutes*, *Bacteroidetes*, *Actinobacteria*, *Proteobacteria*, *Fusobacteria*, and *Verrucomicrobia*, are the most predominant bacterial phyla in adults, with *Firmicutes* and *Bacteroidetes* accounting for 90% of the human gut microbiota.^4^ The composition and abundance of the gut microbiota vary along the longitudinal axis and across the transversal axis of the gastrointestinal (GI) tract. The colon harbors the highest density of microbial cells,^5^ with *Firmicutes* and the *Bacteroides* being mostly represented,^6^ while the small intestine is characterized by bacteria species from the *Streptococcus* genus and *Firmicutes* and *Proteobacteria* phyla.^7^ The gut microbiota also differs along the luminal-mucosa axis.^8^

Bacterial polysaccharides are the main actors of the interaction between bacteria and the external environment. In the gut, these play key roles in bacterial adaptation to the intestinal environment and provide numerous health-promoting benefits to humans, owing to their immunoregulatory properties^9–12^ and various biological activities and physiological functions.^13,14^ Microbial polysaccharides in the gut also come from the consumption of probiotics or from product formulations where they are used for their hydrocolloid techno-functionalities in fermented food (e.g., yogurt and cheese), and pharmaceutical product formulations (e.g., excipients, drug delivery agents).^15,16^

The architecture of the bacterial cell wall differs between Gram-positive and Gram-negative bacteria, as extensively reviewed,^17,18^ and harbors a structurally diverse range of glycans.

**Figure 1.**
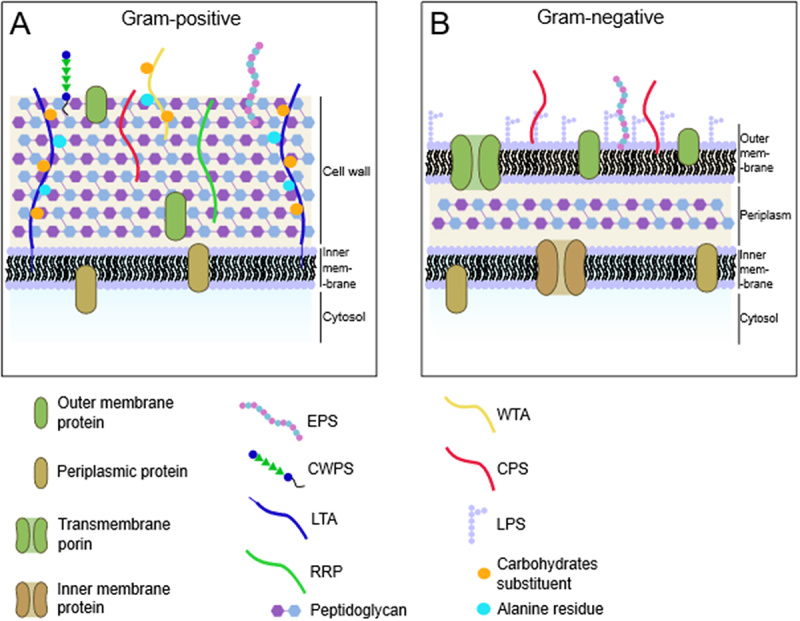
General representation of gram-positive and gram-negative cell wall composition. (a) gram-positive cell wall including variations observed for some ovoid-shaped cocci (*Enterococcus*, *Lactobacillus*, *Lactococcus, Streptococcus* genera, and *Ruminococcus gnavus*) as described in the text; (b) gram-negative organization of the membrane displaying a double lipid bilayer. Image drawn with Inkscape.

Typically, the cytoplasmic membrane of Gram-positive bacteria is covered by a thick layer of peptidoglycan (PG) forming the cell wall (Figure 1(a)). PG is composed of monosaccharide units alternating *N*-acetylglucosamine (GlcNAc) and *N*-acetylmuramic acid (MurNAc), with MurNAc residues further substituted with the so-called peptide-stem containing L- or D- amino acids,^19^ which is cross-linked to another stem via an oligopeptide, whose length depends on the bacterium considered.^20^ PG serves multiple purposes, including bacterial mobility, adherence, and secretion.^21^ Based on its anionic nature, PG is implicated in cation homeostasis,^21^ and plays a crucial role in maintaining the integrity of the bacterial cell membrane under conditions such as low osmolarity. Indeed, mutations of PG biosynthesis genes often lead to cell lysis.^22^ PG can also promote the production of the mucus layer in the gut^23^ and has been implicated in the gut-brain axis, inducing host behavioral changes.^23–25^

The PG layer is crossed with other glycan-containing polymers such as wall teichoic (WTA) and lipoteichoic acids (LTA),^26^ exopolysaccharides (EPS), and/or capsular polysaccharides (CPS) of various carbohydrate compositions, linkages, and molecular weights.^27^ Typically, WTA and LTA structures include a polyol, such as glycerol or ribitol (Rib-ol), phosphodiester units, and additional sugars and differ by the way they are anchored onto the bacterial cell wall.^26,28,29^ CPS and EPS can be homopolysaccharides (HoPS) if made by only one type of monosaccharides, or heteropolysaccharides (HePS) if assembled by different types of monosaccharides with varied linkages.^27^ CPS is tightly attached to the cell surface, often covalently,^30^ whereas EPS is secreted into the extracellular medium.^30^ However, it often occurs that CPS is mistaken as EPS since parts of the capsule can be shed in the medium during *in vitro* bacterial growth. CPS and EPS have been implicated in bacteria – host interactions, specifically by facilitating colonization through their ability to adhere to eukaryotic cells and mucosa, and in microbial-mediated immunomodulation.^31^ In addition, some Gram-positive bacteria species belonging to the species of *Enterococcus, Lactobacillus, Lactococcus, Streptococcus, or Ruminococcus* can carry glycans here referred to as cell wall polysaccharides (CWPS). These consist of a rhamnan-rich polysaccharide (RRP) subunit that, in some cases, is substituted with glycan side chains made of oligosaccharides or polysaccharides, with the latter being referred to as pellicle polysaccharides (PSP).^32^

The cell wall of Gram-negative bacteria, or more precisely Glycobacteria, a certain type of diderm bacteria that are negative to the Gram staining,^33^ is composed of an inner and outer membrane with a thin layer of PG in between them, termed periplasm. The lipopolysaccharide (LPS) constitutes the principal component of the outermost leaflet of the outer membrane, where it covers up to 75% of the total surface (Figure 1(b)). LPS is a large glycolipid composed of three structural domains: lipid A, the core oligosaccharide, and the O-antigen. Lipid A is highly conserved across all LPS characterized so far and consists of a glucosamine disaccharide backbone decorated with 4–6 fatty acid chains and two phosphate units. The lipid A anchors the whole LPS to the outer membrane and is substituted with a branched oligosaccharide domain termed core domain. The O-antigen domain, or O-chain, is a carbohydrate polymer made of several repeating units attached to the core oligosaccharide and comprises the outermost domain of the LPS molecule.^34^ LPS fulfills several functions, such as protecting the bacteria from extracellular factors (e.g., bacteriophages, host antimicrobial peptides, antibiotics), by serving as a physical barrier, or by contributing to the physical properties of the cell.^34^ The role of LPS from members of the gut microbiota has recently been reviewed.^18,35^ Gram-negative bacteria also produce EPS and CPS polysaccharides like Gram-positive bacteria, while, to the best of our knowledge, CWPS and RRP have not been reported in Gram-negative bacteria. In the gut, these polysaccharides are produced by members of the gut microbiota but may also derive from microbes used in fermented foods.

CPS, CWPS, and EPS are implicated in the interaction with the environment of the bacteria and can affect host-bacteria interactions and associated immune responses.^36^ They are often organized in repeating units made of different monosaccharides assembled in a linear or branched manner, resulting in structures of various sizes (from 10^3^ to 10^5^ Da). To date, these cell surface polysaccharides have been extensively studied in the context of pathogen-host interactions due to their role in bacterial pathogenesis.^37^ As a result, bacterial polysaccharides and repeating units from respiratory and enteric pathogens have been immunological targets for the development of antibacterial vaccines.^38^ However, more recent work highlighted cell surface polysaccharides as important factors of the symbiotic interaction between members of the gut microbiota and the host, as traditionally reported for bifidobacteria or lactobacilli species.^15,16,39–42^ While the immunomodulatory properties of polysaccharide capsules from commensal and pathogenic bacteria in the gut are now well acknowledged,^15,16^ detailed information about their chemical composition and structure if often lacking.

Here, we provide an overview of the occurrence, structure, and role of CPS, EPS, and CWPS from commensal or food-derived bacteria found in the gut. We describe the monosaccharides encountered as building blocks of these complex polymers, report the structures determined at molecular level by chemical and spectroscopical approaches, and provide insights into the biological activity of these glycans.

## 2. Occurrence and structures of CPS, EPS, and CWPS from commensal and food-derived bacteria in the gut

### 2.1 Monosaccharide repertoire

In this section, we explore the monomer space, namely the monosaccharide residues, that are assembled for the construction of CPS, CWPS, and EPS in nonpathogenic bacteria found in the gut.

The representation of the monosaccharides, including their stereochemical differences, is reported in Table 1 along with the corresponding symbol (or pictogram) based on the Symbol Nomenclature for Glycans (SNFG).^45^

**Table 1.** Monosaccharides encountered in the glycans produced by nonpathogenic bacteria found in the gut.

L-altruronic acid (L-AltA)	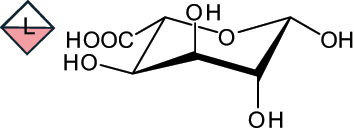	L-arabinose (furanose form) (L-Ara*f*)	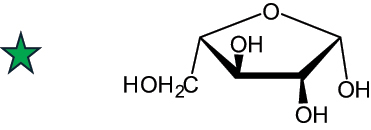
fructose (furanose form) (Fru*f*)	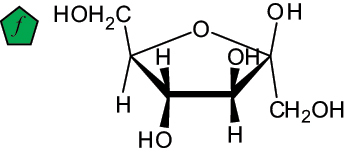	fucose (Fuc)	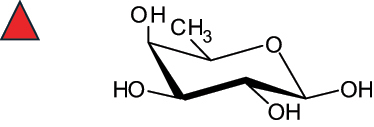
2,4-diamino-2,4-dideoxy-fucose (FucN4N)	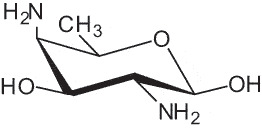	*N*-acetyl-galactosamine (GalNAc)	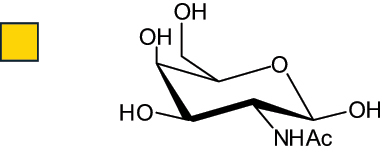
galacturonic acid (GalA)	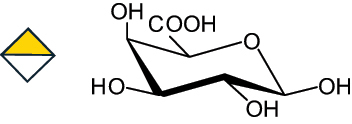	galactose (Gal)	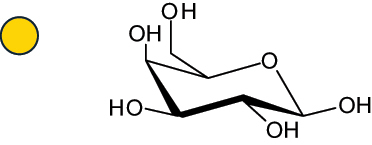
glucosamine (GlcN)	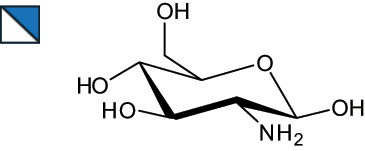	*N*-acetyl-glucosamine (GlcNAc)	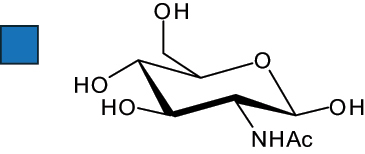
glucuronic acid (GlcA)	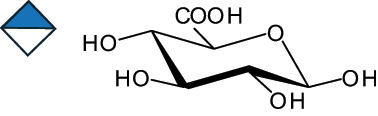	glucose (Glc)	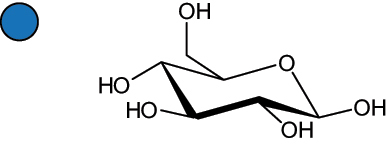
mannose (Man)	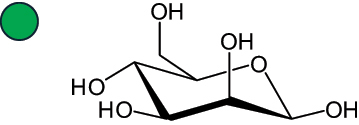	legionaminic acid (Leg)	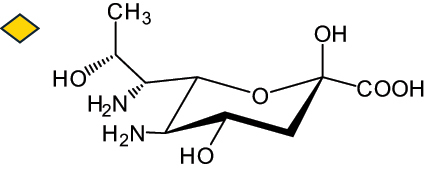
*N*-acetyl-quinovosamine (QuiNAc)	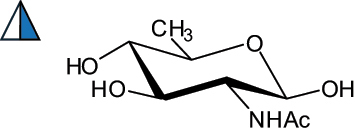	muramic acid (MurA)	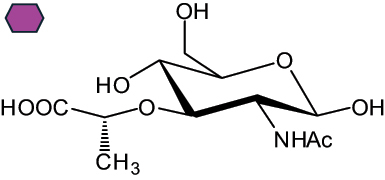
ribose (Rib)	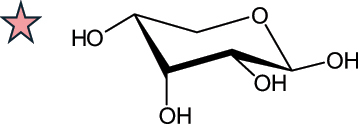	rhamnose (Rha)	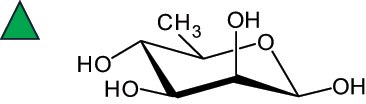
*N*-acetyl-L-rhamnosamine (L-RhaNAc)	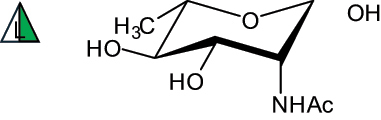	*N*-acetyl-mannosamine (ManNAc)	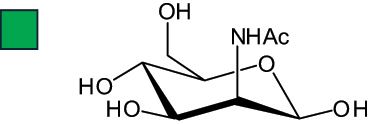
yersinose	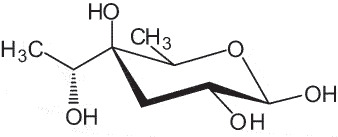 (Yer)	6-deoxy-talose (6dTal)	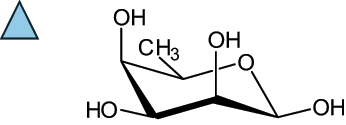
3,9-dideoxy-D-*threo*-D-*altro*-nononic acid with O-2 ether-linked to O-6 of α-D-Glc	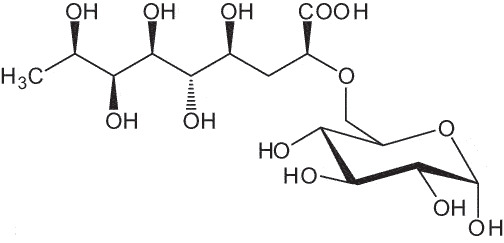	2-keto-3-deoxy-D-*glycero*-D-*galacto*-nononic acid Kdn	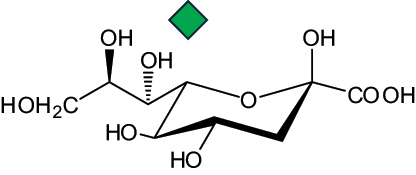

Unless explicitly indicated, the residues of the D series are represented, drawn with the β configuration of the anomeric center and in the pyranose form according to IUPAC rules.^43^ For each residue and where available, the acronym used in the CSDB database is given in brackets along with the corresponding symbol.^44^.

To date, the total number of SNFG symbols available is 75, ten of which are relevant to human glycans. However, this is not sufficient to cover the diversity encountered from all types of bacterial glycans (about 100).^18^ Here, we show that CPS, EPS, and CWPS from commensal or food-derived bacteria in the gut rely on the assembly of 24 residues, with the most common monosaccharides being neutral, such as galactose (Gal), glucose (Glc) and rhamnose (Rha), as described in the following sections. The only positively charged residue at physiological pH is 2,4-diamino-2,4-dideoxy-fucose (FucN4N) since its amino function at position 4 is in the free form while the one at position 2 is acetylated. Negatively charged monosaccharides belong to the class of uronic acids, namely under the form of altruronic acid (AltA), galacturonic acid (GalA), glucuronic acid (GlcA), legionaminic acid (Leg), and 2-keto-3-deoxy-D-*glycero*-D-*galacto*-nononic acid (Kdn) (Table 1). In addition to these charged residues, the overall charge of glycans can be influenced by the presence of phosphate groups or lactic acid (see section 2.3.1). These moieties are part of the possible decorations, such as acetyl groups, which, in most cases, are *N*-linked to the amino function of aminosugars, as detailed in the following Tables, which report the full structures of the glycans (Tables 2–6). Finally, a rather rare decoration occurs when the alditol of a monosaccharide is ether-linked to another residue, like in the case of the Glc residue with the alditol 3,9-dideoxy-D-*threo*-D-*altro*-nononic acid, deriving from a nonulosonic acid, which is ether-linked to O-6 of the Glc unit.^146^

**Table 2.** Structure of the glycans characterized from gram-negative bacteria found in the gut.

Name	Structure	Type	CSDB	Ref
*A. xylinum* B42	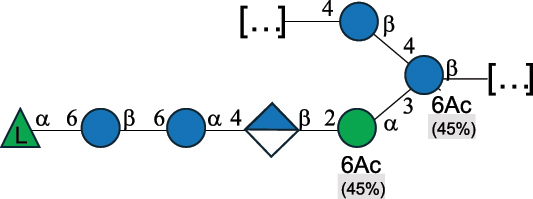	CPS	3792	Ojinnaka et al.^46^
*B. fragilis* 23745M1	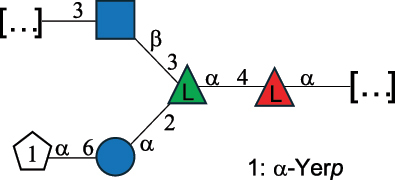	CPS	4024	Pavliak et al.^47^
*B. fragilis* NCTC 9343	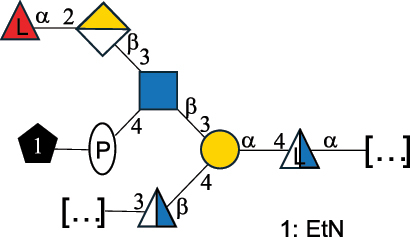	CPS or EPS CPS-B	107777	Baumann et al.^48^
*B. fragilis* NCTC 9343	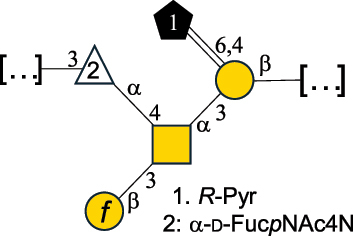	CPS or EPS CPS-A	29289 107776	Surayot et al.^42^ Toukach et al.^44^ Mostafavi et al.^49^ Baumann et al.^48^
*B. vulgatus* IMCJ1204	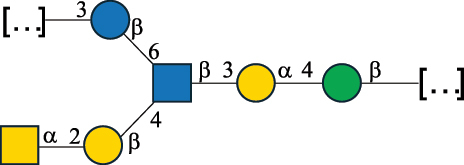	CPS	500	Hashimoto et al.^50^

“*A*.” stands for Acetobacter, and “*B*.” stands for *Bacteroides*, the Carbohydrate Structure Database (CSDB)^44,51,52^ identification number is given in the corresponding column. The structures are depicted according to the rules given by the Systematic Nomenclature of Glycans. Where not specified, all the residues are in the pyranose form (if furanose, a “*f*” is reported inside the symbol). All residues have the D absolute configuration unless otherwise specified. The detailed chemical structure, along with the symbols used, is presented in Table 1. The symbol “[…]” indicates the extremes of the repeating unit.

**Table 3.** Polysaccharides (EPS, CPS, and CWPS) structurally characterized from *Enterococcus* species found in the gut.

Name	Structure	Type	CSDB	Ref
*E. faecalis* VE14089, FA2–2	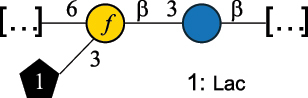	CPS-C	3691 26495	Guerardel et al.^53^ Theilacker et al.^54^
*E. faecalis* V583	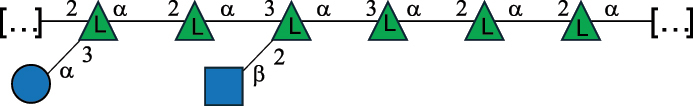	CWPS*	32112	Theilacker et al.^55^ Guerardel et al.^53^
*E. faecium* Tx16	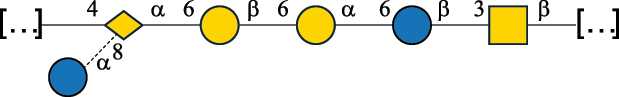	EPS	30965	Kodali et al.^56^
*E. faecium* Tx16	[levan 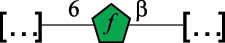	EPS	30718	Kodali et al.^56^
*E. faecium* Tx16	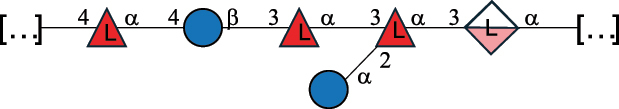	EPS	30959	Kodali et al.^56^
*E. faecium U0317*	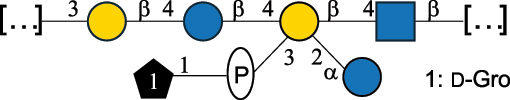	CPS	26148	Laverde et al.^57^

“*E*.” stands for *Enterococcus*, and the CSDB identification number is shown in the appropriate column.^44,51,52^ The structures are depicted according to the rules given by the Systematic Nomenclature of Glycans. Where not specified, all the residues are in the pyranose form (if furanose, a “*f*” is reported inside the symbol) and have the D absolute configuration. “P” in an ellipse stands for phosphate. Dotted linkages indicate a non-stoichiometric substituent. The monosaccharide chemical structure, along with the symbols used, is in Table 1. The symbol “[…]” indicates the extremes of the repeating unit.

*This polysaccharide is also referred to as enterococcal polysaccharide antigen, EPA.

**Table 4.** Polysaccharides (EPS, CPS, PS, and CWPS) structurally characterized from *Lactobacillus (lb.), Lacticaseibacillus (Lcb.), Leuconostoc (Leu.)*, *Pediococcus (P.)*, and *Weissella (W.)* species found in the gut.

Name	Structure	Type	CSDB	Ref
*Lb. acidophilus* sp. 5e2	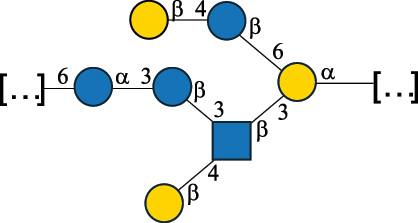	EPS	29710	Patten et al.^58^
*Lb. acidophilus* LMG 9433	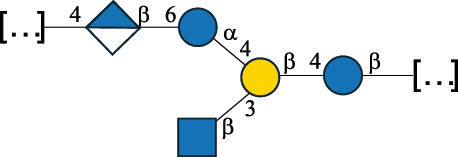	EPS	4353	Robijn et al.^59^
*Lb. casei* BL23	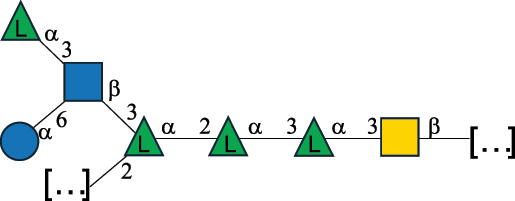	CWPS	11862	Vinogradov et al.^60^
*Lb. casei* BL23	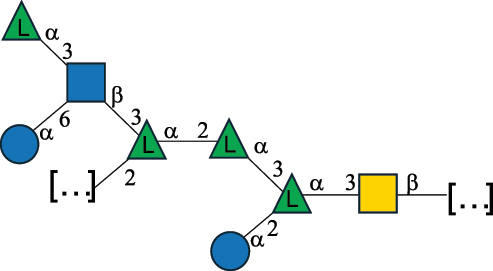	CWPS	11863	Vinogradov et al.^60^
*Lb. casei* LC2W	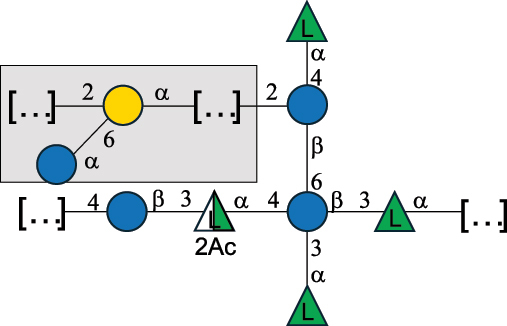	EPS*	1168	Ai et al.^61^
*Lb. casei* LOCK 0919	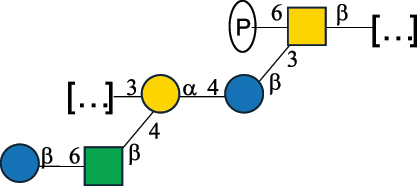	PS	11899	Gorska et al.^62^
*Lb. delbrueckii* ssp. bulgaricus 17	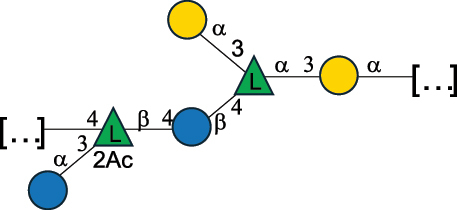	PS	30736	Vinogradov et al.^63^
*Lb. delbrueckii* ssp. bulgaricus 17	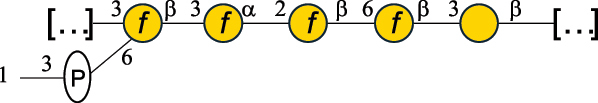	PS	30980	Vinogradov et al.^63^
*Lb. delbrueckii* ssp. bulgaricus 24, 25, Lfi5 (NCC556), LY03, NCC15, rr	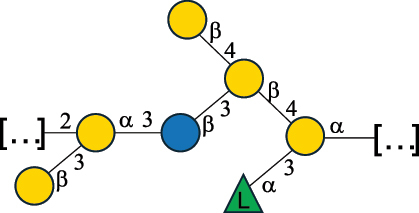	EPS	11223 117090	Marshall et al.^64^ Lamothe et al.^65^ Van Calsteren et al.^66^ Gruter et al.^67^
*Lb. delbrueckii* ssp. bulgaricus 291	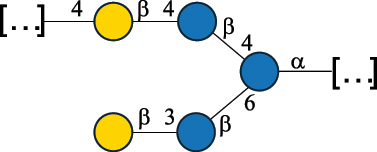	CWPS	11222	Van Calsteren et al.^66^
*Lb. delbrueckii* ssp. bulgaricus EU23	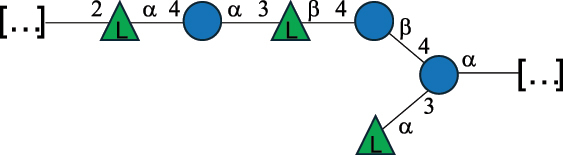	EPS	11221 481	Van Calsteren et al.^66^ Harding et al.^68^
*Lb. delbrueckii* ssp. bulgaricus LBB.B26	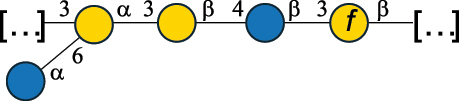	EPS	21724 11225	Van Calsteren et al.^66^ Sanchez-Medina et al.^69^
*Lb. delbrueckii* ssp. bulgaricus LBB.B332	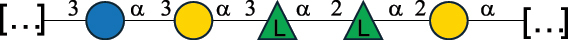	CWPS	11226	Van Calsteren et al.^66^
*Lb. delbrueckii* ssp. bulgaricus NCFB2074	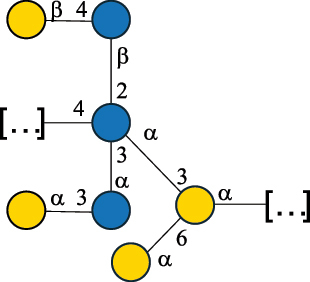	EPS	11224	Van Calsteren et al.^66^ Harding et al.^70^
*Lb. delbrueckii* ssp. bulgaricus OLL1073R–1	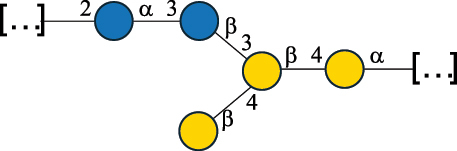	CWPS	11078	Van Calsteren et al.^66^
*Lb. delbrueckii* ssp. bulgaricus SRFM-1	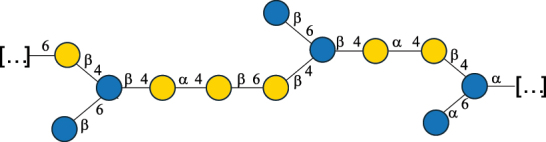	EPS	12041 3221	Tang et al.^71^ Tang et al.^72^
*Lb. farciminis* CIP 103136	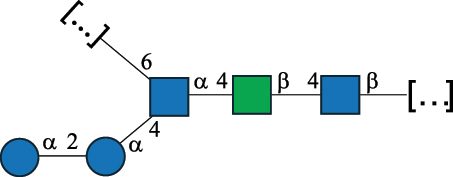	CPS	1967	Maes et al.^73^
*Lb. fermentum* Lf2	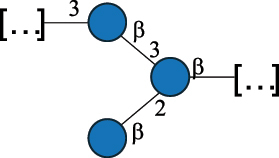	EPS	1134	Vitlic et al.^74^
*Lb. fermentum* Lf2	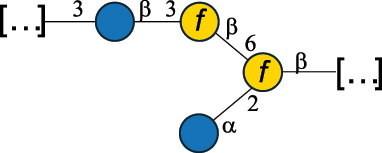	EPS	3254	Ahmed et al.^75^
*Lb. fermentum* Lf2	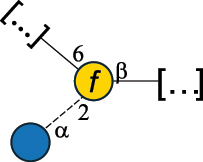	EPS	3255	Ahmed et al.^75^
*Lb. fermentum* MTCC 25067	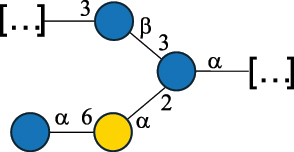	EPS	731	Gerwig et al.^76^
*Lb. fermentum* TDS030603	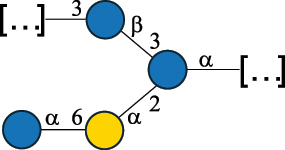	EPS	29180	Gerwig et al.^77^
*Lb. helveticus*	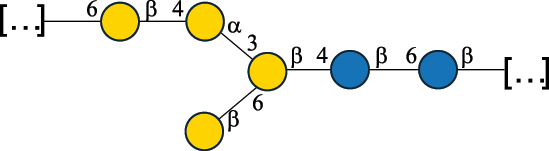	EPS	4935	Staaf et al.^78^
*Lb. helveticus* DPC4571	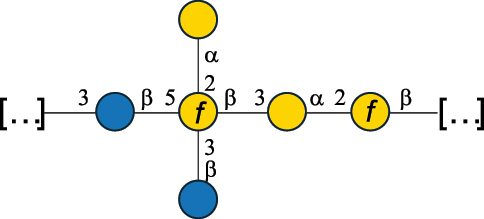	CWPS	29377	Vinogradov et al.^79^
*Lb. helveticus* K16	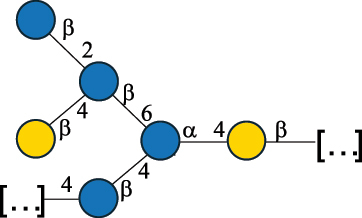	EPS	5923	Yang et al.^80^
*Lb. helveticus* Lb161	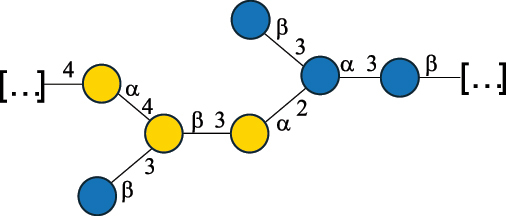	EPS	21724	Staaf et al.^81^
*Lb. helveticus* LH1	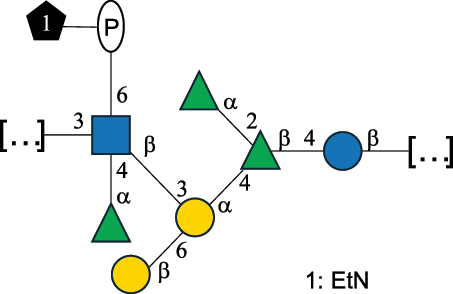	CWPS	29793	Vinogradov et al.^79^
*Lb. helveticus* LH1	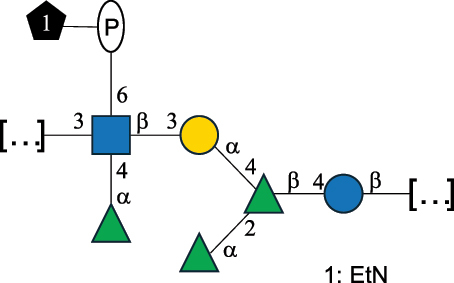	CWPS	29792	Vinogradov et al.^79^
*Lb. helveticus* Lh59, TN-4	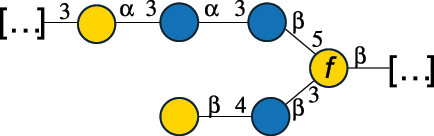	EPS	4957 5905	Stingele et al.^82^ Yamamoto et al.^83^
*Lb. helveticus* LZ-R-5	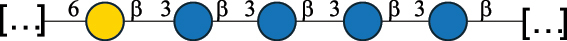	EPS	33240	You et al.^84^
*Lb. helveticus* MB2–1	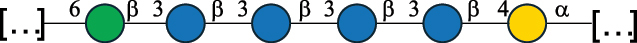	EPS	7684	You et al.^85^
*Lb. helveticus* MB2–1	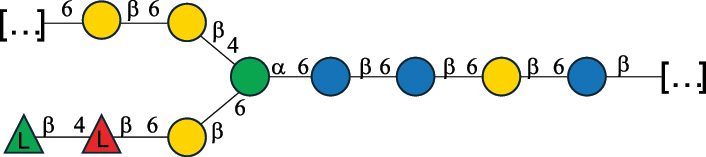	EPS	8466	Ge et al.^86^
*Lb. helveticus* sp. Rosyjski	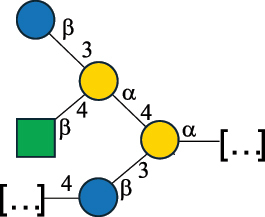	EPS	29309	Patten et al.^58^
*Lb. helveticus* SNA12	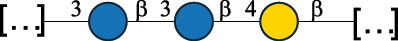	EPS	8568	Wang et al.^87^
*Lb. helveticus* TY1–2	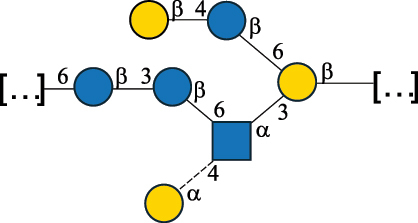	PS	6006 118066	Yamamoto et al.^83^ Yamamoto et al.^88^
*Lb. kefiranofaciens*	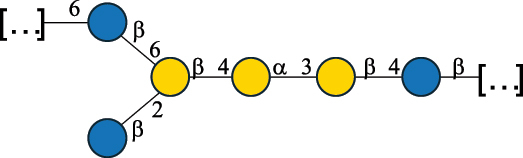	EPS	43912	Ghasemlou et al.^89^
*Lb. lactis* ssp. cremoris B891	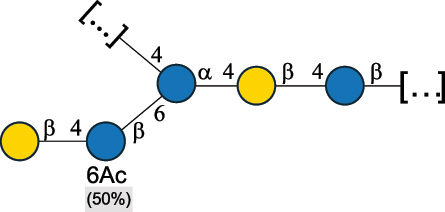	EPS	5263 5317	van Casteren et al.^90^
*Lb. mucosae* VG1	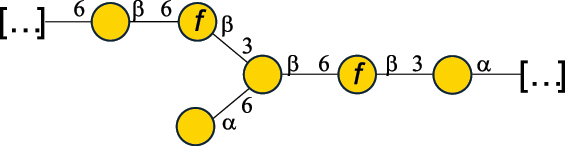	EPS	1007	Fagunwa et al.^91^
*Lb. paracasei* 34–1	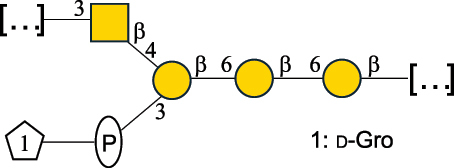	EPS	4354	Robijn et al.^92^
*Lb. paracasei* 34–1	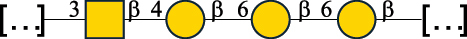	EPS	4483	Robijn et al.^92^
*Lb. paracasei* DG	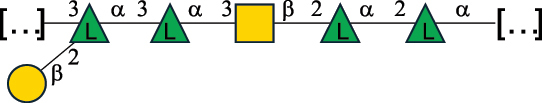	EPS	12014	Balzaretti et al.^93^
*Lb. pentosus* LPS26	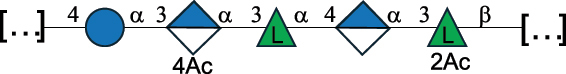	EPS	22725	Rodriguez-Carvajal et al.^94^
*Lb. pentosus* LZ-R-17	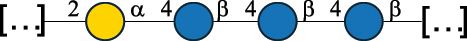	EPS	32239	You et al.^85^
*Lb. plantarum* AR307	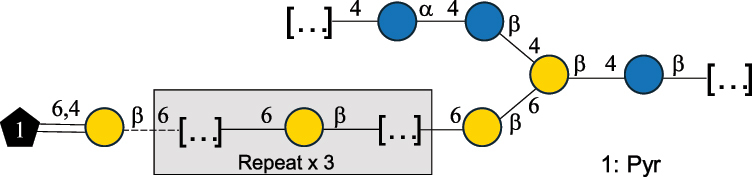	EPS*	10847	Feng et al.^95^
*Lb. plantarum* IMB19	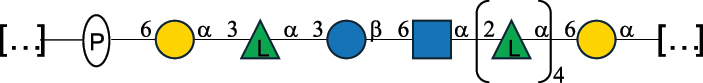	CPWS	32106	Garcia-Vello et al.^96^
*Lb. plantarum* C70	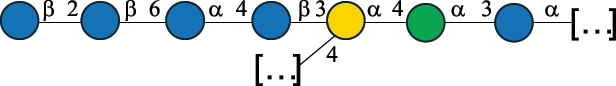	EPS	32058	Ayyash et al.^97^
*Lb. plantarum* C88	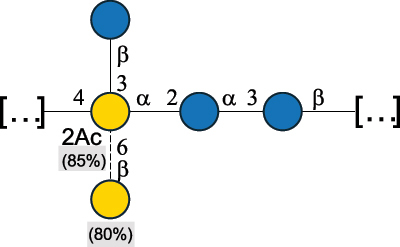	EPS	30599 30755 30756	Fontana et al.^98^
*Lb. reuteri* 180	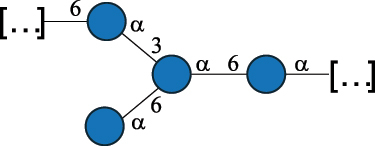	EPS	10950	Gerwig^76^
*Lb. rhamnosus* ATCC 53103	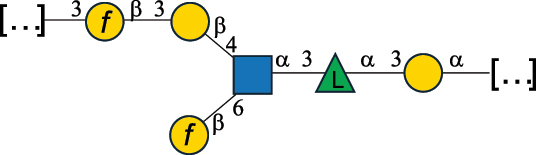	EPS	3114	Landersjo et al.^99^
*Lb. rhamnosus* BIM B-1039	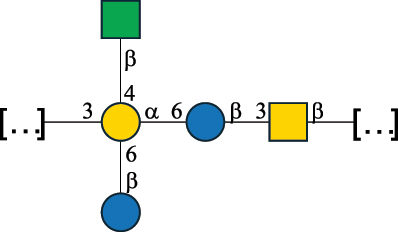	EPS	1156	Zdorovenko et al.^100^
*Lb. rhamnosus* BIM B-1039; . *casei* Lb31; *Lcb. rhamnosus* RW-9595 M, KL37D, R, E/N	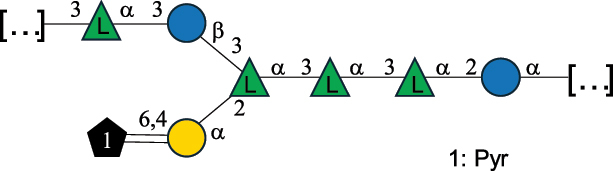	EPS	2361 25068	Zdorovenko et al.^100^ Knirel et al.^101^
*Lb. rhamnosus* C83, KL37A, KL37C, KL37D	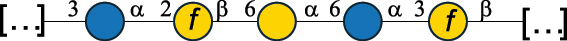	EPS	5274 3164	Vanhaverbeke et al.^102^ Lipinski et al.^103^
*Lb. rhamnosus* KL37B	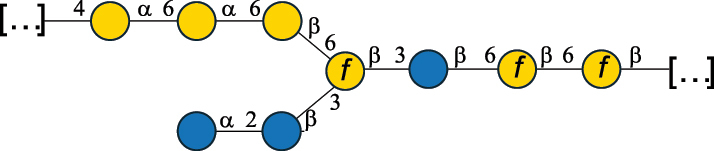	EPS	26208	Gorska-Fraczek et al.^104^
*Lb. rhamnosus* LOCK 0900	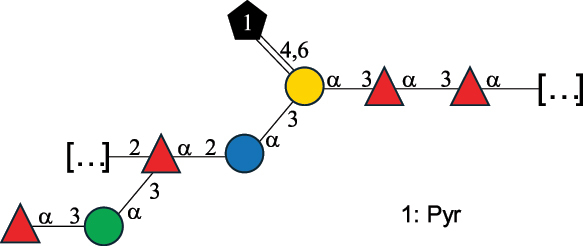	CWPS	15050	Gorska et al.^105^
*Lb. rhamnosus* LOCK 0900	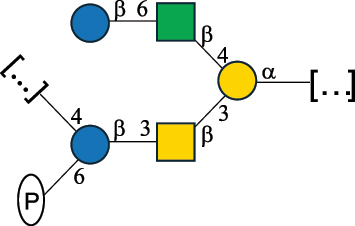	CWPS	15051	Gorska et al.^105^
*Lb. sake* 0–1	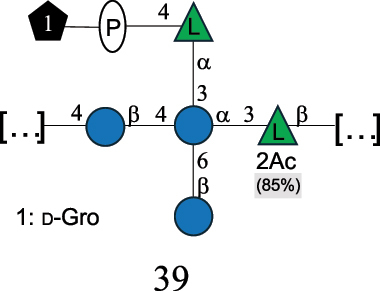	EPS	25077	Knirel et al.^101^
*Lcb. casei* RW-3703 M	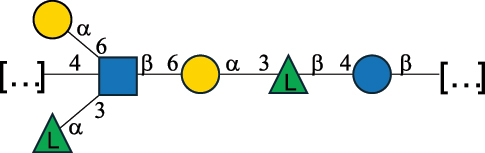	EPS**	-	Van Calsteren et al.^106^
*Lcb. paracasei* NPB01	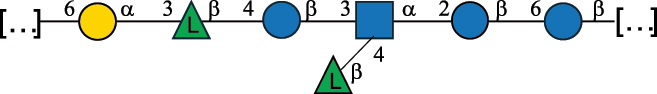	CPS-1**	-	Armiento et al.^107^
*Lcb. paracasei* NPB01	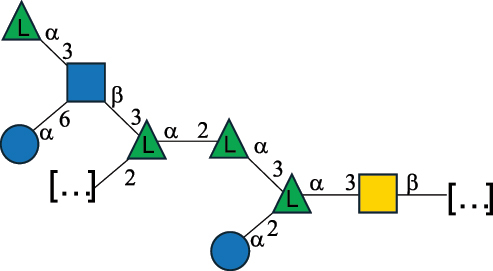	CPS-2**	-	Armiento et al.^107^
*Lcb. paracasei* Shirota YIT 9029	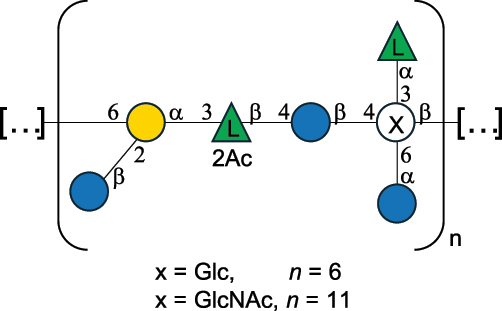	CPS*	21071	Mizukoshi et al.^108^
*Lcb. paracasei* ZY-1	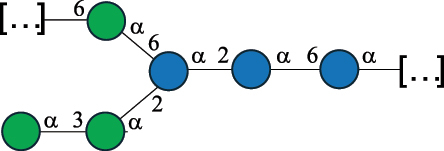	EPS	8574	Xiao et al.^109^
*Lcb. plantarum* 70810	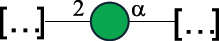	EPS	9787***	Tian et al.^110^
*Lcb. plantarum* 70810	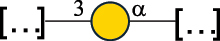	EPS	9788***	Tian et al.^110^
*Lcb. plantarum* 70810	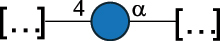	EPS	10950***	Tian et al.^110^
*Leu. mesenteroides* ssp. cremoris PIA2	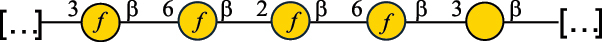	CPS	26359	Svensson et al.^111^
*P. damnosus* 2.6	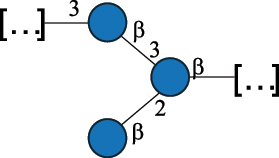	EPS	2023	Duenas-Chasco et al.^112^
*P. pentosaceus* M41	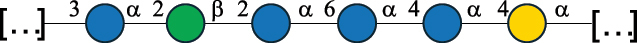	EPS	32056	Ayyash et al.^113^
*W. confusa* KR780676	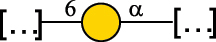	EPS	27074	Zhang et al.^114^

The CSDB identification numbers are listed in the table. The structures are depicted according to the rules given by the Systematic Nomenclature of Glycans.^44,51,52^ Where not specified, all residues are in the pyranose form (if furanose, a “*f*” is reported inside the symbol). All residues have the D absolute configuration unless otherwise specified. Dotted linkages indicate a non-stoichiometric substituent. The detailed chemical structure, along with the symbols used, is presented in Table 1. The symbol “[…]” indicates the extremes of the repeating unit.

*The repeats in the shaded boxes are the motifs attached to the main glycan backbone.

**The structure is not on the CSDB website at the time this review was written.

***Assigned to the *Lacticaseibacillus* genus in the original publication.

**Table 5.** Polysaccharides (EPS, PSP, RRP, and CWPS) structurally characterized from the *Lactococcus* species found in the gut.

Name	Structure	Type	CSDB	Ref
*Lc. garvieae* C47	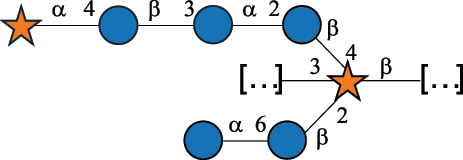	EPS	32057	Ayyash et al.^115^
*Lc. lactis* 1196	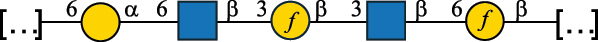	C-type PSP	5151	Mahony et al.^32^
*Lc. lactis* 1196, 3107, JM1, MG1363, W34	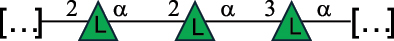	C-type RRP	5144	Mahony et al.^32^
*Lc. lactis* 3107	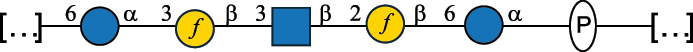	C-type PSP	5149	Mahony et al.^32^
*Lc. lactis* A76	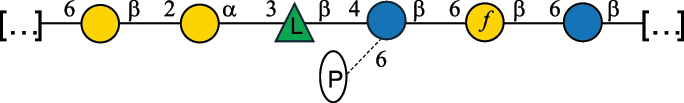	C-type PSP	5153	Mahony et al.^32^
*Lc. lactis* BIM B-1024	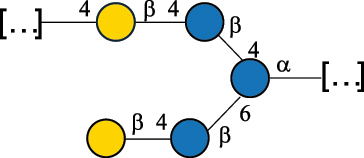	EPS	11991	Zdorovenko et al.^116^
*Lc. lactis* BIM B-1024	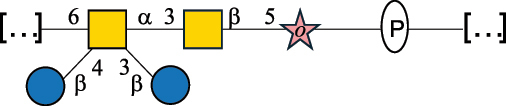	EPS	12331	Zdorovenko et al.^116^
*Lc. lactis* JM1	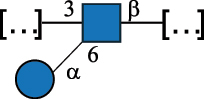	C-type PSP	5147	Mahony et al.^32^
*Lc. lactis* LL-2A	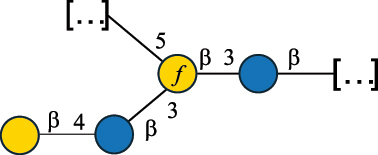	EPS	5181	Nachtigall et al.^117^
*Lc. lactis* MG1363	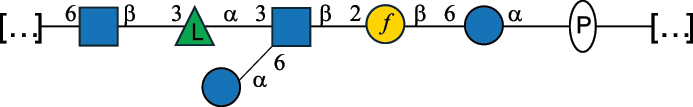	C-type PSP	25204	Mahony et al.^32^ Chapot-Chartier et al.^118^
*Lc. lactis* SK11	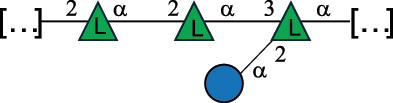	C-type RRP	5145	Mahony et al.^32^
*Lc. lactis* SK11	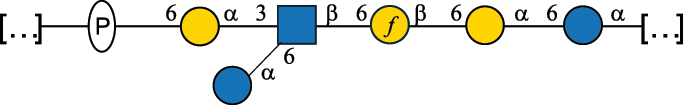	C-type PSP	5146	Mahony et al.^32^
*Lc. lactis* SMQ-388	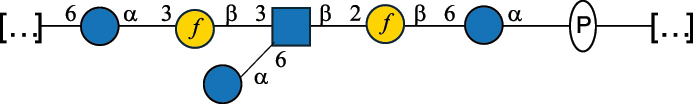	C-type PSP	5148	Mahony et al.^32^
*Lc. lactis* W34	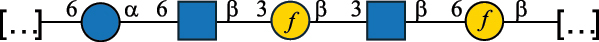	C-type PSP	5150	Mahony et al.^32^
*Lc. lactis* UC509.9	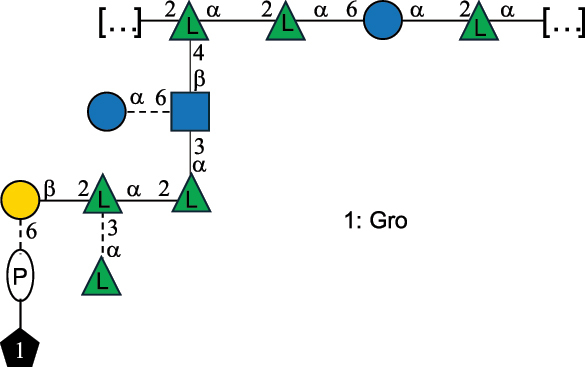	A-type CWPS	5155	Mahony et al.^32^
*Lc. lactis* ssp. cremoris B39	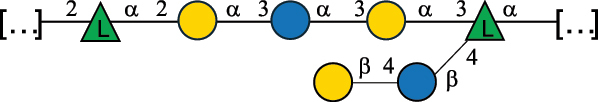	EPS	5262	van Casteren et al.^90^
*Lc. lactis* ssp. cremoris B40, SBT 0495	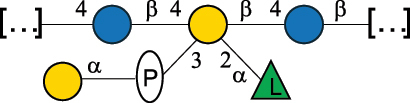	EPS	8394	Knirel et al.^101^ van Casteren et al.^119^
*Lc. lactis* cremoris H414	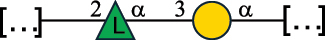	RRP part of CWPS*	22415	Gruter et al.^120^
*Lc. lactis* cremoris H414	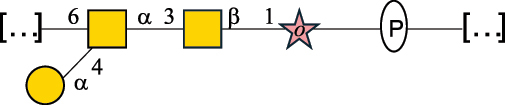	PSP part of CWPS*	22472	Gruter et al.^120^
*Lc. lactis* ssp. lactis 184	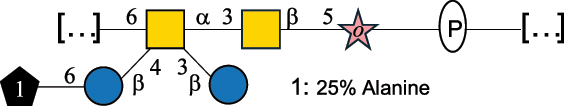	D-type PSP	5154	Mahony et al.^32^
*Lc. lactis* ssp. lactis IL1403	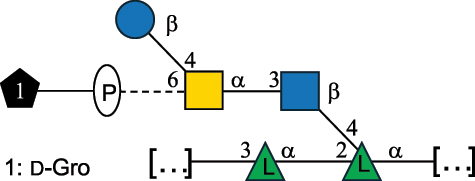	B-type CWPS	12811	Mahony et al.^32^ Vinogradov et al.^121^
*Lc. lactis* ssp. lactis IO-1	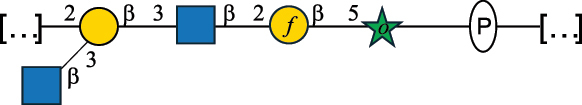	C-type PSP	5152	Mahony et al.^32^

“*Lc*.” stands for *Lactococcus*, and the CSDB identification number is listed in the table.^44,51,52^ The genotype (e.g., A-type) is indicated for all the *Lc*. strains for which this information is available. The structures are depicted according to the rules given by the Systematic Nomenclature of Glycans. Where not specified, all the residues are in the pyranose form (if furanose, a “*f*” is reported inside the symbol, alditols are denoted with “*o*”) “P” in an ellipse stands for phosphate. All residues have the D absolute configuration unless otherwise specified. Dotted linkages indicate a non-stoichiometric substituent. The monosaccharide chemical structure, along with the symbols used, is in Table 1. The symbol “[…]” indicates the extremes of the repeating unit.

*not confirmed.

**Table 6.** Polysaccharides (EPS, PSP, RRP, and CWPS) structurally characterized from nonpathogenic *Streptococcus* and *Ruminococcus* species found in the gut.

Name	Structure	Type	CSDB	Ref
*R. gnavus* ATCC 35193	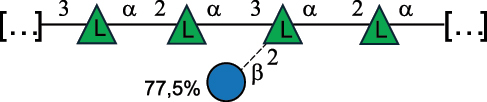	EPS*	-	Laplanche et al.^122^
*R. gnavus* ATCC 29149	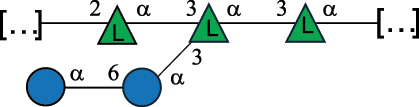	EPS and CWPS	10941	Henke et al.^123^
*R. gnavus* E1	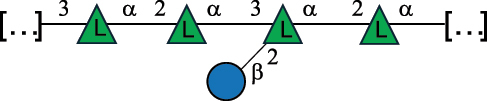	EPS*	-	Laplanche et al.^122^
*S. macedonicus* Scl36	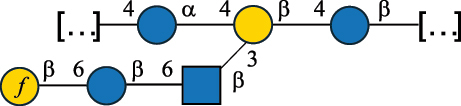	EPS	5295	Vincent et al.^124^
*S. macedonicus* Scl36	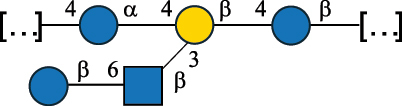	EPS	5319	Vincent et al.^124^
*S. salivarius* ssp thermophilus strain NCBF 2393	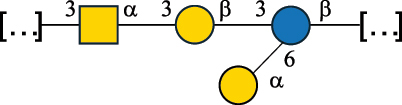	EPS	6865	Griffin et al.^125^ Doco et al.^126^
*S. thermophilus* 21, IMDO1, IMDO2, IMDO3, NCFB 859	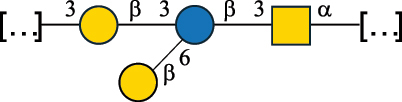	EPS	3513	Marshall et al.^64^
*S. thermophilus* 8S	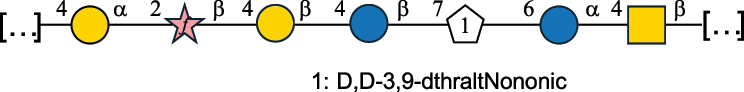	EPS**	2050	Faber et al.^127^
*S. thermophilus* strains AR333, S-3	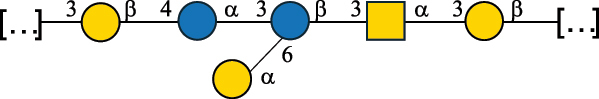	EPS	10969 13013	Zhang et al.^128^ Xu et al.^129^
*S. thermophilus* ASCC 1275	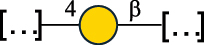	EPS	5186	Padmanabhan et al.^130^
*S. thermophilus* ASCC 1275	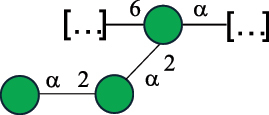	EPS	5188	Padmanabhan et al.^130^
*S. thermophilus* ASCC 1275, STCth-9204, SY89, SY102, SFi39	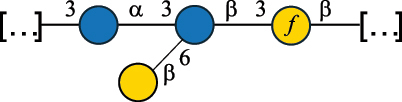	EPS	32173 12385 3407 10408	Marshall et al.^64^ Padmanabhan et al.^130^ Pachekrepapol et al.^131^ Germond et al.^132^ Lemoine et al.^133^
*S. thermophilus* CS6	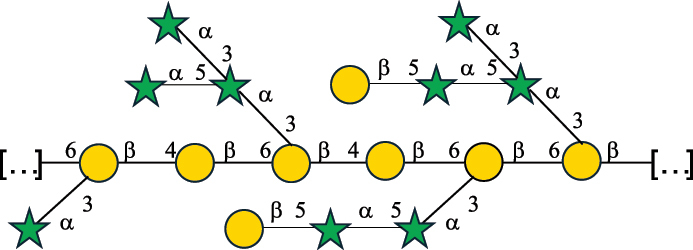	EPS***	10982	Zhou et al.^134^
*S. thermophilus* DGCC 7698, EU20	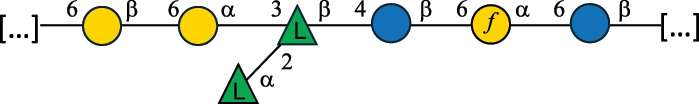	EPS	12007 3408	Pachekrepapol et al.^131^ Marshall et al.^135^
*S. thermophilus* DGCC 7710	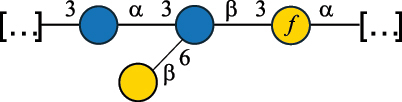	EPS	12381	Pachekrepapol et al.^131^
*S. thermophilus* DGCC 7785	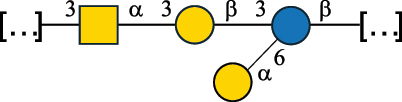	EPS	12382	Pachekrepapol et al.^131^
*S. thermophilus* DGCC7919	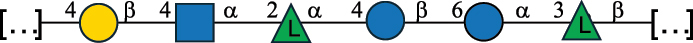	EPS	32168	Nachtigall et al.^117^
*S. thermophilus* GST-6, ST1	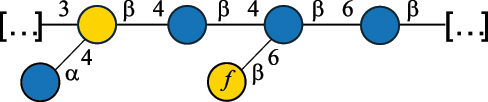	EPS	11352	Zhang et al.^136^ Säwén et al.^137^
*S. thermophilus* OR901, Rs, Sts	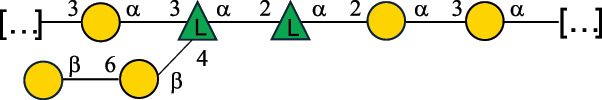	EPS	3130	Li et al.^138^ Leeflang et al.^139^
*S. thermophilus* OR901, RS, *Sts*	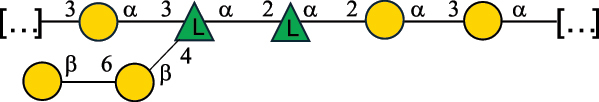	EPS	10411	Leeflang et al.^139^
*S. thermophilus* S3	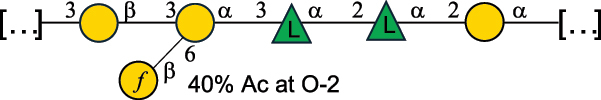	EPS	10413	Faber et al.^140^
*S. thermophilus* SFi12	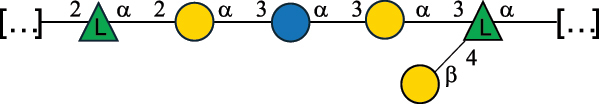	EPS	10409	Lemoine et al.^133^
*S. thermophilus* SFi20	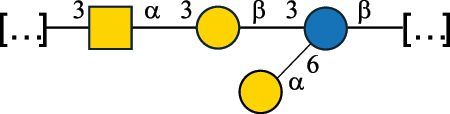	EPS	10412	Navarini et al.^141^
*S. thermophilus* ST1	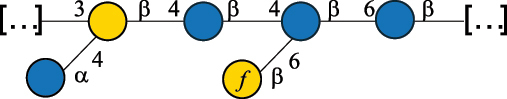	EPS	10406	Säwénet al.^137^
*S. thermophilus* ST-143	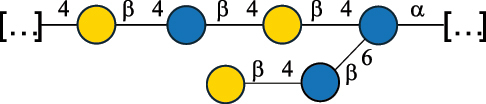	EPS	12384	Pachekrepapol et al.^131^
*S. thermophilus* ST-10255y, ST 4239	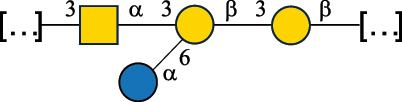	EPS	12383	Pachekrepapol et al.^131^
*S. thermophilus* ST64987	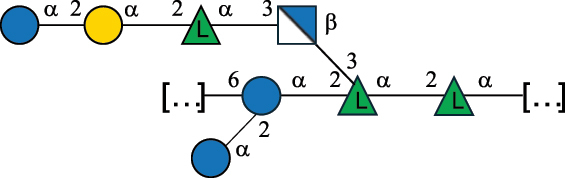	CWPS	5160	McDonnell et al.^142^
*S. thermophilus* ST64987	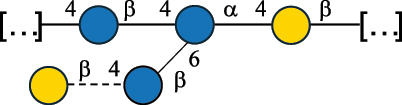	EPS	5163	McDonnell et al.^142^
*S. thermophilus* THS	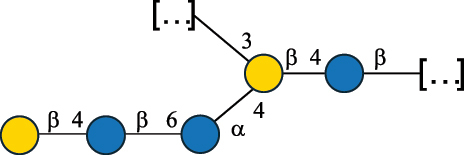	EPS	10407	Nordmark et al.^143^
*S. thermophilus* UCCSt12	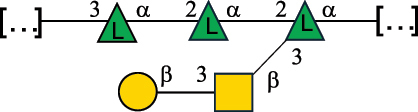	CWPS	21042	Lavelle et al.^144^
*S. thermophilus* UCCSt89	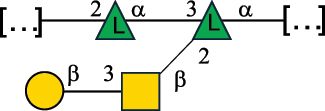	CWPS	21039	Lavelle et al.^144^
*S. thermophilus* UCCSt89	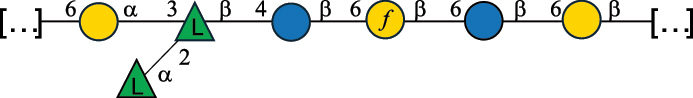	EPS	21041	Lavelle et al.^144^
*S. thermophilus* UCCSt95	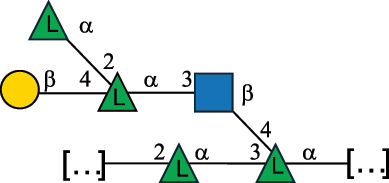	CWPS	8495	Lavelle et al.^144^
*S. thermophilus* ZJUIDS-2–01	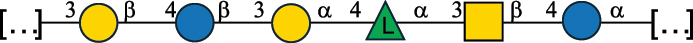	EPS	108032	Cao et al.^145^

“*R*.” stands for *Ruminococcus*, and “*S*.” for *Streptococcus*, the CSDB identification number is reported in the table,^44,51,52^ if available. The structures are depicted according to the rules given by the Systematic Nomenclature of Glycans. Where not specified, all the residues are in the pyranose form (if furanose, a “*f*” is reported inside the symbol), “P” in an ellipse stands for phosphate. All residues have the D absolute configuration unless otherwise specified. Dotted linkages indicate a non-stoichiometric substituent. The monosaccharide chemical structure, along with the symbols used, is in Table 1. The symbol “[…]” indicates the extremes of the repeating unit.

*The structure is not on the CSDB website at the time this review was written.

**the nonulosonic acid is linked in open-form as acetal.

***All arabinose (Ara) units are L and in the furanose form.

### 2.2. CPS and EPS from non-pathogenic gram-negative bacteria in the gut

Most CPS/EPS structures of Gram-negative bacteria in the gut have been identified and characterized from members of the *Bacteroidota* and *Proteobacteria* phyla, namely *Escherichia coli* and *Bacteroides* species.

*E. coli* (in the phylum *Proteobacteria*) is a gut commensal bacterium, but this species also comprises several pathogenic strains which have been the focus of thorough investigations. As a result, a total of 80 different CPS structures, classified according to their genetic and biochemical data, have been described.^147^ These structures are available from a public repository at www.iith.ac.in/EK3D/ and, therefore, are not described here. Most of these CPS are released in the growth medium and, therefore, sometimes referred to as EPS. This includes EPS from the probiotic strain *E. coli* Nissle 1917 (EcN), which is a heparosan, K5 in the serological classification.^148^

The CPS structures from several *Bacteroides* species (in the *Bacteroidota* phylum) have been determined, including those from *B. fragilis*^48,49^ and *B. vulgatus*^50^ strains (see Table 2).

*B. fragilis* NCTC 9343 produces two different capsules (labeled CPS-A or CPS-B),^48^ both with a branched repeating unit (Table 2). CPS-A includes the rare sugar α-D-Fuc*p*NAc4N and a 4,6-pyruvate Gal, while CPS-B is composed of two *N*-acetyl-quinovosamine (QuiNAc) residues (with both D- and L-absolute configuration), a GalA residue, and a glucosamine (GlcN) linked at the O-4 position with a 2-aminoethylphosphonate group.^48^ Similarly, CPS from *B. fragilis* 23745M1 contains an uncommon sugar as a terminal unit of its side chain named yersinose (Yer; Table 1). These features confer *Bacteroides* CPS a neutral or zwitterionic character (displaying moieties with opposite charges). Although *B. thetaiotaomicron* VPI-5482 (ATCC-29148D) has been shown to produce phase-variable CPS and lipoproteins^149–151^ and can synthesize Kdn (structure in Table 1),^152^ the structure of its CPS has not been characterized.

Also included here is the CPS structure of *Acetobacter xylinum* B42, as this Gram-negative species is used in the fermentation of drinks like beer^46^ (Table 2). From a structural point of view, it consists of a β-(1→4)-Glc backbone substituted on alternate Glc residues with a pentasaccharide side chain, made of Rha, Glc, GlcA and mannose (Man) units.

### 2.3. CPS, CWPS, and EPS from non-pathogenic firmicutes (gram-positive bacteria) in the gut

The interest in the cell wall polysaccharides of bacteria belonging to the Firmicutes phylum has borne several decades ago from the study of industrially relevant bacteria named lactic acid bacteria (LAB), including *Lacticaseibacillus*, *Leuconostoc*, *Pediococcus*, *Lactococcus*, *Streptococcus*, *Enterococcus* and *Weissella*. Of note, the *Lactobacillus* species named *casei*, *paracasei* and *rhamnosus* have been recently reclassified into a new genus, *Lacticaseibacillus*,^153^ but the structures reported in the next Tables adopt the name given in the original publication.

All these bacteria are mostly found in food and have been suggested to exert many health-promoting functions in the gut and contribute to the shaping of a healthy microbiota by influencing host-microbe and microbe-microbe interactions.^154,155^ Most of these bacteria produce different types of glycans, with those secreted having interesting texturizing properties, responsible for the “ropy” phenotype of the bacterial colonies. According to a recent review, *Leuconostoc*, *Weissella*, and *Pediococcus* mostly produced HoPS, whereas the remaining genera were the main HePS producers.^41^ When these glycans are inserted in the cell wall, they are referred to as CWPS,^55^ and in most cases, they consist of two domains, the first is linked to the muramic acid of the PG layer and is characterized by the prevalence of Rha residues, here referred to as RRP. Of note, in bacteria covered with CWPS, teichoic acids are only present in small amounts or absent.^55^

The RRP domain can be substituted with glycan side chains, whose nature is species/strain specific and is named PSP in *Lactococcus* species.^32^

The *Lactobacillus* genus^79^ also harbors different glycans referred to as WPS (cell wall polysaccharide) and/or NPS (neutral polysaccharide) to differentiate them from the anionic teichoic acids. In this review, these glycans will all be termed CWPS to minimize the confusion between the various terms/acronyms used in different studies.

The structures of CWPS from *Enterococcus*, *Lactobacillus*, *Lactococcus*, *Streptococcus* and *Ruminococcus* are reported separately in the next sections. *Weissella, Pediococcus* and *Leuconostoc*, mostly produce dextran- and/or fructan-like glycans, which are not included in this review because their sources and use has been recently reviewed elsewhere.^156–158^ For these bacteria, only the glycans with a structure different from these two polysaccharides will be reported.

#### 2.3.1. The enterococcal glycans

*Enterococci*, originally classified as members of the *Streptococcus* genus, produce EPS, CPS, while their RRP often does not have the additional appendages which is expected of a complete CWPS (Table 3).

*Enterococci* are considered part of the human gut microbiota, with *E. faecalis* and *E. faecium* being the most representative species, although these have been proposed as opportunistic pathogens, contributing to urinary tract infections, bacteremia, bacterial endocarditis, diverticulitis, and meningitis, while displaying resistance to several antibiotics.^159^ Therefore, there has been a focus on glycan analyses of these two species to develop glycan-based vaccines to prevent infections.

Almost 60% of *E. faecalis* strains have been classified into four different serotypes (labeled A to D) based on the reaction of their putative CPS with opsonic antibodies, even though at the time, the structures of the capsules were not known.^54^ Today, CPS-A has been identified as a teichoic acid,^160^ the structures of CPS-B and CPS-D remain to be defined, and the CPS-C structure of two *E. faecalis* strains has been characterized,^53–55^ as reported in Table 3.

*E. faecium* encodes glycans of complex structures with no resemblance to any other RRP-like architecture. *E. faecium* T×16 strain harbors three distinct glycans on the cell surface,^56^ one assembled by many units of β-(2→6) fructofuranose (namely a levan), and the other two, both HePS, presenting quite uncommon monosaccharide units (reported in Table 1): L-altruronic acid (L-AltA) and legionaminic acid (Leg), a nonulosonic acid often associated with human pathogenic bacteria such as *Acinetobacter baumannii*.^161^ All these glycans were used to raise protective antibodies, and the glycoconjugates with the two HePS displayed the desired activity while the glycoconjugate with levan did not.^56^ Similar results were observed for the CPS from *E. faecium* U0317, showing that *E. faecium* glycans (apart from levan) can be immunogenic if conjugated to a suitable carrier protein. However, information about their role in the gut environment is unknown.

#### 2.3.2. The glycans from the lactobacillaceae family

The *Lactobacillaceae* family is divided into 25 genera due to their extremely high genotypic, phenotypic and ecological diversity.^153^ *Lactobacillus* (*Lb*.), *Leuconostoc* (*Leu*.), *Pediococcus* (*P*.) and *Weissella* (*W*.) genera are all part of this family.^162^ Importantly, many species originally classified within the *Lactobacillus* genus have been reclassified.^153^ Indeed, the species *casei*, *paracasei*, *pentosus*, and *rhamnosus* are now part of the *Lacticaseibacillus* genus, the species *plantarum* is in the new genus *Lactiplantibacillus* (*Lcp*.), the species *fermentum*, *mucosae*, and *reuteri* are in the new genus *Limosilactobacillus*, the species *farminicis* is in the new genus *Companilactobacillus*, and the species *sake* has been renamed *sakei* and together with *curvatus* is part of the *Latilactobacillus genus*.^153^

For clarity, the bacterial names in Table 4 refer to those used in the original publications and this review will use the term *Lactobacillus* to refer to these bacteria.

*Lactobacillus* species can be used as starter cultures in the production of fermented milk products. For instance, *Lb. delbrueckii* subsp. *bulgaricus* and *Lb. delbrueckii* sbsp. *lactis* are used with *Streptococcus thermophilus* in the production of Italian hard cheeses.^163^ Some other species, like *Lb. plantarum*, *Lb. casei*, *Lb. paracasei*, *Lb. rhamnosus*, and *Lb. curvatus*, are found in fermented milk products, where they proliferate during the maturation of the product, thus contributing to its final flavor.^164^ In addition, some Lactobacillus species are autochthonous members of the human gut microbiota but in different proportion depending on the population origin.^165^

The cell wall in *Lactobacillus* sp. is covered with CPS and EPS and other types of glycans,^32,55^ whose classification did not follow that adopted for *Enterococci*, *Lactococci*, and *Streptococci* for which the acronyms CWPS, RRPs, and PSP are used. Indeed, *Lactobacillus* glycans have been named differently, such as: i) NPS, namely glycans that are attached (or assumed to be attached) to the PG layer and that are neutral, ii) WPS, which are similar to NPS but with an ionic character, or more generally iii) polysaccharides, covering glycans closely or loosely associated with the bacterial surface. In the following sections, we extend the definition of CWPS to include NPS and WPS, since both are considered anchored to the PG of the bacterial cell wall, while EPS or CPS classification will be used as introduced above. Finally, and in contrast to other *Firmicutes*, *Lactobacilli* can have an additional outer layer, termed S-layer, made of protein subunits assembled together, sometimes glycosylated,^166,167^ for which the glycan structural part is still poorly defined.

From a structural point of view, Gal and Glc are the main constituents of *Lactobacillaceae* polysaccharides analyzed to date (Table 4), with 37.7% and 35% abundance compared to the total monosaccharide content, respectively, followed by 17% L-Rha (of which 15.6% refers to L-Rha and 1.4% to D-Rha). Of note, Glc is always in the pyranose form, while Gal is found both in pyranose (30.7%) and furanose (7%) forms. When in the furanose form, Gal is β-configured at the anomeric center, except for the polysaccharide from *Lb. delbrueckii* ssp. *bulgaricus* 17 strain in which one of the four galactofuranose residues is α-configured.^63^ L-Rha is present in all *Lactobacillus* and *Lacticaseibacillus* polysaccharides apart from those produced by *Lb. helveticus* LH1.^79^ Hexosamines are less abundant, only contributing to 5.9% of the total monosaccharide content, whereby GlcNAc and GalNAc are most abundant, accounting for 3% and 1.8%, respectively, of the overall monosaccharide composition. Interestingly, GlcA was the only uronic acid monosaccharide found, representing 0.7% of the total monosaccharide content. Finally, some chemical modifications were reported, such as *O*-acetylation, pyruvate linked as a ketal at the O-4 and O-6 positions of terminal galactopyranose, glycerol phosphate, and EtN phosphate found as side chain substituents in several CPS.^79,101^

#### 2.3.3. The Lactococcal glycans

*Lactococcus* (*Lc*.), originally part of the *Streptococcus* genus, includes *Lc. lactis*, a bacterium widely used for the production of fermented food, especially buttermilk and cheese.

*Lc. lactis* has been classified into four genotypes based on the genetic profile of the cell wall polysaccharide gene cluster (*cwps*) of more than hundred strains.^32^ The C-type CWPS present, for example, on *L. lactis* strain MG1363 is the prevalent one (Table 5); it is characterized by a linear rhamnan (the RRP domain), decorated with sidechains consisting of a regular polysaccharide (PSP), which nature determines CWPS further classification into subtypes (e.g. C_1a_, C_1b_, C_2_).

The A- and B-types present more heterogeneous and complex CWPS. The A-type (present, for example, in *Lc. lactis* UC509.9 strain) is characterized by a RRP linear backbone made of three Rha and one Glc unit, with every third unit in the sequence being substituted with a branched oligosaccharide containing a phosphate in addition to other substituents in non-stoichiometric proportions (Table 5). The RRP of the B-type (present, for example, in *Lc. lactis* IL1403 strain) consists of a linear backbone with a α-Rha disaccharide repeating unit, irregularly substituted with a trisaccharide that occasionally carries a glycerol (Gro) moiety linked via a phosphodiester linkage (Table 5). For the CWPS D-type (present, for example, in *Lc. lactis* 184 strain), genetic analyses suggested a different type of RRP, but its structure is not available yet, while the structure of the PSP has no resemblance to others (Table 5). Since only one D-type strain has been analyzed to date, no general rules about its CWPS structural features can be established yet.

The structure of *Lc. garvieae* EPS has been included in Table 5 since, while being a recognized fish pathogen, *Lc. garvieae* strain C47 has been reported as a potential prebiotic, and its EPS is considered a potential thickener of fermented camel milk.^115^ Its EPS structure shows a highly branched repeating unit composed of a 3-linked β-xylose as linear backbone on which two side chains are linked: a disaccharide composed of α-Glc-(1→6)-β-Glc and a tetrasaccharide consisting of a terminal xylose and three glucose units, linked at position 2 and 4 of the xylose, respectively.

Based on the monosaccharide content of polysaccharides produced by *Lactococcus* bacteria whose structures have been determined so far, D-Glc, D-Gal, and L-Rha appear to be the main constituents, with an abundance of 27.1%, 25% (14.7% for pyranose and 10.3% for furanose form), and 24.2%, respectively. Interestingly, a deep inspection of the structures revealed that Rha is always α-configured at the anomeric center (except in *Lc. lactis* A76,^32^ and 2- or 3-linked rather than in terminal location; with 2-Rha being most represented (63% of the total Rha content). Gal can be found in α-or β-configuration at the anomeric center for the pyranose form, whereas galactofuranose is always β-configured. Regarding hexosamines, GlcNAc (12.5% abundance) is mostly present as β-6, β-3 or β-3,6 linked; while GalNAc (5.1% abundance) is α-6 or β-3 linked. Of note, polyols represent 4.4% of the monosaccharides content (2.2% Rib-ol, 1.5% Gro and 0.7% arabinitol or Ara-ol). These polysaccharides are essentially neutral or negatively charged due to the presence of a phosphate group.

#### 2.3.4. The Streptococcal glycans

*Streptococci* (*S*.) cover many species, most of them known as pathogens of the oral or respiratory tract, such as *S. pneumoniae*, the causative agent of pneumonia, *S. pyogenes*, which causes pharyngitis, or *S. mutans*, which is implicated in dental caries.

There is a large structural diversity in the type of glycans decorating the *Streptococcus* cell wall. For example, *S. pneumoniae* produces WTA, LTA, and CPS, with about 100 different structures determined to date^168,169^ but lacks the CWPS, which, in contrast, is present in *S. thermophilus*, *S. agalactiae*, *S. dysgalactiae*, *S. mutants*, and *S. pyogenes*.^55^

The only species relevant to the gut is *S. thermophilus*, formerly known as *S. salivarius* subsp. *thermophilus*. This bacterium is used in the fermentation of milk to yogurt in combination with *Lactobacillus delbrueckii* subsp. Bulgaricus; while *S. macedonicus* is used in the production of goat cheese.^124^

*S. thermophilus* strains generally produce branched EPS and/or CWPS (Table 6) with one or two side chains, each made by one to four monosaccharide residues, except for *S. thermophilus* 8S,^127^ ASCC 1275,^130^ and ZJUIDS-2–01^145^ strains, which have a linear EPS. *S. thermophilus* glycans are mainly composed of Gal (~46% abundance, of which 88% are in pyranose form and 12% in furanose form), followed by Glc (26% abundance) and Rha (16% abundance). Of note, arabinofuranose (Ara*f*) is only found in the EPS produced by *S. thermophilus* CS6.^134^ Interestingly, the EPS from *S. thermophilus* 8S strain contains atypical components, namely D-Rib*f* and the alditol 3,9-dideoxy-D-*threo*-D-*altro*-nononic acid, whose O-2 is ether-linked to O-6 of a α-Glc unit (Table 1).^127,146^ On the other hand, *S. macedonicus*^124^ and *S. salivarius* strains^125^ produce branched EPS composed of: Glc (47% abundance), Gal (33% abundance), GlcNAc (13% abundance), and GalNAc (7% abundance). As in *S. thermophilus*, Gal is the only unit found in both pyranose and furanose forms. Regarding the hexosamine content, only β-6-GlcNAc and α-3-GalNAc have been reported in *Streptococcal* glycans (Table 6).

#### 2.3.5. The Ruminococcus gnavus glycans

*R. gnavus* belongs to the *Clostridiales* order and *Lachnospiraceae* family within the phylum of the *Firmicutes*. *R. gnavus* is a prevalent member of the human gut microbiota in infants and adults and is associated to health and disease, as recently reviewed.^170^ While retaining its name from the original classification in the genus *Ruminoccocus*, *R. gnavus* has been temporarily classified into the genus *Blautia* and more recently into the genus *Mediterraneibacter*.^171^ This bacterium produces a RRP that appears to be anchored to the muramic residue of the PG layer via a phosphodiester linkage.^123^ This RRP is also released in the growth medium as EPS, and a thorough NMR investigation has surmised the presence of additional appendages made by poly-glycerol-phosphate.^172^ *R. gnavus* type strain ATCC 29,149 produces a glucorhamnan referred to as glucorhamnan-I composed of a Rha backbone made of 2- and 3-linked Rha units, and a side chain consisting of the disaccharide α-Glc-(1→6)-α-Glc (Table 6).^123^ *R. gnavus* ATCC 35,913 and E1 strains produce a glucorhamnan named glucorhamnan-II, whose repeating unit has a backbone made of four β-L-Rha units, with alternating 2- and 3-linkages, and a side chain consisting of a β-D-Glc residue linked to the O-2 position of the 3-Rha (Table 6).^122^ These two EPS show variation in the glucosylation level that is non-stoichiometric in *R. gnavus* ATCC 35193, along with subtle variations in their immunological properties (see section 3.2).^122^

### 2.4. EPS, WPS, and CPS from actinobacteria in the gut

The *Actinomycetota* (Actinobacteria) phylum covers *Bifidobacterium* (*Bf*.) and *Propionibacterium* (*Pr*.) strains for which EPS structures are available (Table 7).^187^

**Table 7.** Polysaccharides (EPS, WPS, and CPS) structurally characterized from actinobacteria species found in the gut.

Name	Structure	Type	CSDB	Ref
*Bf. adolescentis* YIT 4011	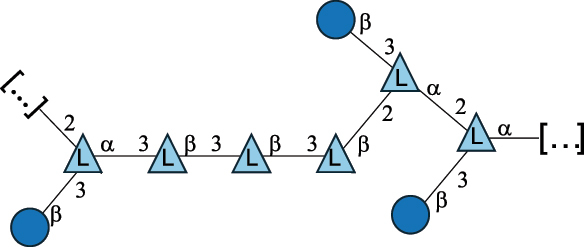	CPS	132385 30261 6468	Nagaoka et al.^173^ Hidalgo-Cantabrana et al.^174^ Pyclik et al.^175^
*Bf. animalis* ssp. lactis LKM512	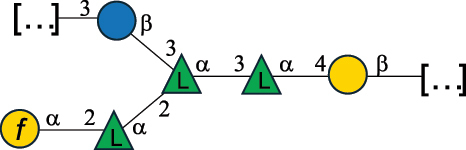	EPS	6467	Pyclik et al.^175^ Uemura et al.^176^
*Bf. animalis* ssp. lactis RH	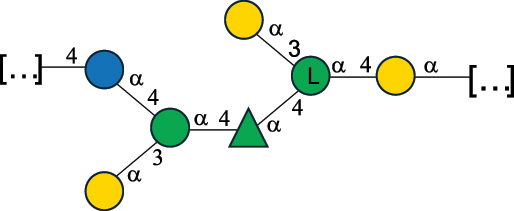	EPS	30260 6466	Hidalgo-Cantabrana et al.^174^ Pyclik et al.^175^
*Bf. bifidum* BIM B-465	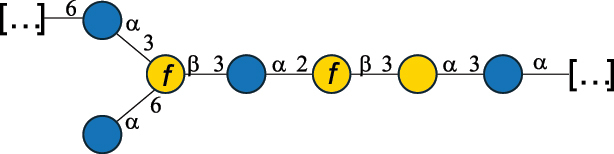	EPS	30262 6469 23890	Hidalgo-Cantabrana et al.^174^ Pyclik et al.^175^ Zdorovenko et al.^177^
*Bf. bifidum* PRI1	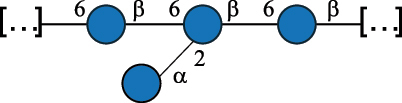	CPS (CSGG)	6470	Speciale et al.^178^ Verma et al.^179^
*Bf. bifidum* PRI1	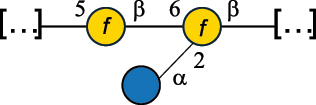	CPS (CSGG)	6471	Speciale et al.^178^ Verma et al.^179^
*Bf. bifidum* PRI1	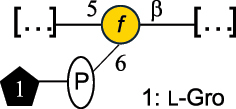	CPS (PGbG)	6460	Speciale et al.^178^ Verma et al.^179^
*Bf. bifidum* PRI1	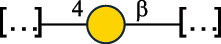	CPS (CSGG)	6474	Speciale et al.^178^ Verma et al.^179^
*Bf. bifidum* PRI1	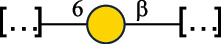	CPS (CSGG)	6473	Speciale et al.^178^ Verma et al.^179^
*Bf. breve* YIT 4007	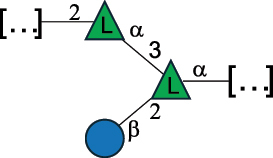	WPS	132364	Habu et al.^180^
*Bf. breve* YIT 4010	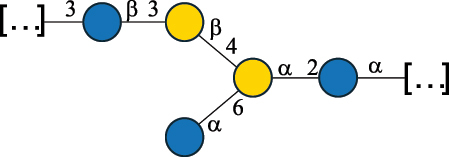	EPS	132365 30263	Hidalgo-Cantabrana et al.^174^ Habu et al.^180^
*Bf. infantis* Reuter ATCC 15697	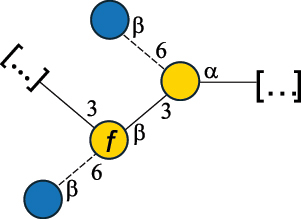	CPS	5215	Tone-Shimokawa et al.^181^
*Bf. longum* BIM B-476-D	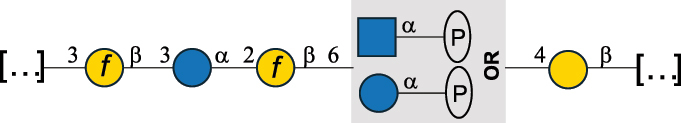	EPS	29781	Valueva et al.^182^
*Bf. longum* JBL05	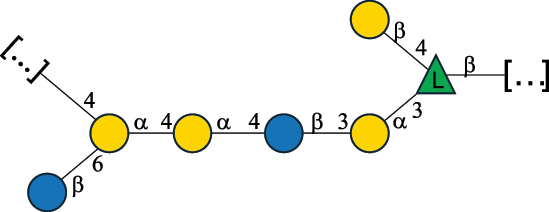	EPS	25101 30267 6479	Knirel et al.^101^ Hidalgo-Cantabrana et al.^174^ Pyclik et al.^175^ Kohno et al.^183^
*Bf. longum* YIT 4028	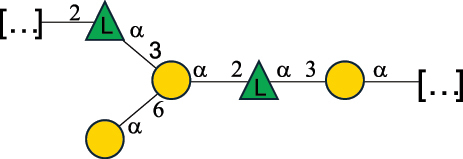	EPS	30266 3742	Hidalgo-Cantabrana et al.^174^ Nagaoka et al.^184^
*Bf. longum* W11	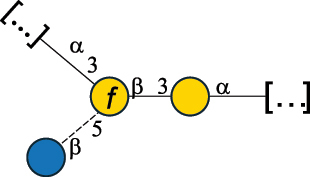	CPS	12037	Inturri et al.^185^
*Bf. longum* ssp. longum 35624	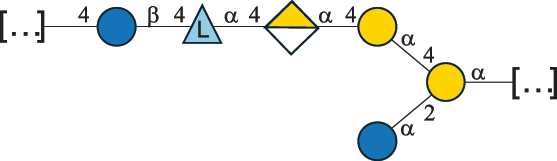	EPS	11848	Altmann et al.^186^
*Pr. freudenreichii* 109, 111; *P. thoenii* 126	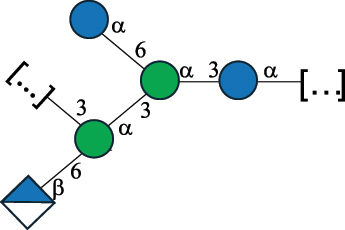	EPS	22636	Dobruchowska et al.^187^

“*Bf*.” stands for *Bifidobacterium*, and *Pr*.” stands for *Propionibacterium*. The CSDB identification number is in brackets. The structures are depicted according to the rules given by the Systematic Nomenclature of Glycans. Where not specified, all residues are in the pyranose form (if furanose, a “*f*” is reported inside the symbol). All residues have the D absolute configuration unless otherwise specified. Dotted linkages indicate a non-stoichiometric substituent. The detailed chemical structure, along with the symbols used, is presented in Table 1. The symbol “[…]” indicates the extremes of the repeating unit.

*Bifidobacteria* were discovered in infant feces in 1900, and the name reflects the characteristic Y-shape morphology of the bacterium. These bacteria, originally named *Bacillus bifidus*, were later included in the *Lactobacillus* genus, and then (in 1974) moved to a separate genus within the *Actinomycetales*.^188^

*Bifidobacteria* produce glycans assumed to be attached to the PG layer, although the chemical linkage to the muramic acid unit has not been determined.^175^ These glycans share many of the building blocks found in other gut bacteria, namely Gal, Glc, and Rha, with Gal and Glc making up to 36.7% of the total monosaccharide content, followed by Rha at 11.4%. Glc is only found in the pyranose form, whereas Gal is found both in the pyranose and furanose forms, with a preference for the pyranose form (72.5% of total Gal). These glycans can be neutral or exhibit a net negative charge due to the presence of phosphate groups, as shown for *Bf. longum* BIM B-476-D,^182^ or due to the presence of the acidic monosaccharide, GalA, as shown for *Bf. longum* ssp. longum 35624 (Table 7). Similarly, *Bf. bifidum* cell surface is covered with glycans of predominant neutral character, as shown for *Bf. bifidum* BIM-465 and PRI1 strains which are composed of neutral hexoses, Gal and Glc (Table 7). Of note, in *Bf. bifidum* PRI1, the cell surface is covered by five types of glycans, four being neutral and named cell surface β-glucan/galactan (CSGG), and one having an anionic character due to the presence of a phosphate and named polysaccharide phosphoglycero-β-galactofuran (PGβG).^178,179^ Given the location of these glycans on the bacterial surface, these are here classified as CPS.

In contrast to other gut bacteria, the EPS from some Bifidobacteria also contains 6-deoxy-L-talose (6dTal)^173–175,186^ (Table 7), accounting for 8.9% of the total monosaccharide content. 6dTal is a rare monosaccharide, whose occurrence in Gram-negative bacteria is limited to EPS and LPS of a few members of the *Burkholderia*, *Rhizobium*, and *Yokenella* genera.^44^ Of note, the genus that most embeds this monosaccharide into its cell wall is *Mycobacterium*, genetically related to *Bifidobacterium* as found in the same phylum.

The EPS structure of *Propionibacterium* (*Pr*.) has been characterized from three *Pr. freudenreichii* probiotic strains,^187^ all producing the same GlcA-containing anionic glycan (Table 7).

## 3. Biological roles of CPS, EPS, and CWPS from nonpathogenic bacteria in the gut

Cell surface polysaccharides covering CPS, CWPS, EPS, RRP, and PSP are composed of oligosaccharide repeating units, often including acidic moieties such as uronic acids or phosphates, which are often directly anchored to the PG layer on the cell surface of bacteria or loosely associated with it.^189^ These different types of polysaccharides, extending above the bacterial surface, are essential for the interaction of bacteria with the environment and maintaining the dynamic balance of intestinal microbial communities. The cell wall polysaccharides of members of the human gut microbiota have mainly been studied for their role in conferring immune tolerance and promoting gut homeostasis through their direct impact on immune cells or via modulation of the gut microbiome structure and function, while the biological effects of EPS from probiotics and food-derived LAB have been investigated for their prebiotic, antioxidant, anticancer, and immunomodulation activities against intestinal infections, as recently reviewed.^15^

### 3.1. Biological roles of microbial CPS in the gut

CPS composition varies among strains and serotypes of bacterial species.^189^ Although mainly studied in pathogens,^37^ these polysaccharides have been shown to play important roles in the interaction between gut commensal bacteria and the host, where they protect bacteria from host and environmental factors and influence host immunity.^14,190^ The best studied examples of CPS in gut commensal microbes are from *B. fragilis* and *B. thetaiotaomicron* strains. *B. fragilis* NCTC 9343 CPS-A, which is composed of 2-acetamido-4-amino-2,4,6-trideoxygalactose, Gal, and GalNAc^48^ (Table 2), is also named polysaccharide A (PSA) in biological studies. PSA anti-inflammatory effects occur through the induction of IL-10 by CD4+ T cells.^14,191^ PSA can bind to the TLR1/2 heterodimer, leading to the modulation of Treg.^192,193^ One of the CPS produced by *B. fragilis* NTCT 9343 has been shown to activate CD4^+^ T cell expression by presentation through the MHC-II complex^194^ and to activate the intestinal sensory neurons.^195^ Of note, the condition necessary for the MHC-II to display CPS requires them to be zwitterionic, which is a property also recurring in other bacteria.^194^ *B. thetaiotaomicron* VPI-5482 (or ATCC-29148D) CPS capsule is composed of GlcNAc, Glc, Man and GalA but no other structural data are available to date.^13^ *B. thetaiotaomicron* CPS can be either pro-stimulatory or anti-stimulatory, resulting in inhibiting antigen (Ag) delivery to the immune system or activating Ag-specific T cell response.^14^

At the genomic level, CPS from *Bacteroidetes* are encoded by diverse *cps* biosynthetic loci as shown in *B. thetaiotaomicron*,^13^ *B. fragilis*,^196,197^ and in the human gut microbiome,^198^ underscoring the importance of CPS diversity to the fitness of bacteria that inhabit the gut. Indeed, the diversity of CPS harbored by species of the gut microbiota enables the modulation of adaptive immune responses to their dominant antigens by altering CPS expression.^14,150^

These CPS can also be phase variable. For example, the gut symbiont *B. thetaiotaomicron* VPI-5482 expresses eight different CPS to adapt to various niches such as immune, bacteriophage, and antibiotic perturbations.^13,14,150^ The ability of *B. thetaiotaomicron* VPI-5482 to switch between multiple capsules confers increased fitness in the mouse gut.^13^ This diversity in CPS expression is a testament to the long-term adaptation of gut microbiota species to evade host immunity by altering the microbial patterns that may otherwise be recognized by the host.

CPS from Gram-positive gut bacteria have also been investigated for their role in modulating host immune response. For example, *Faecalibacterium prausnitzii* strain HTF-F, belonging to the *Firmicutes* phylum and one of the most abundant species in healthy human colon, underrepresented in the microbiota of IBD patients, produces a CPS (of which composition and structure are unknown) but with demonstrated anti-inflammatory immunomodulatory properties.^199^ In addition, the probiotic strain *E. coli* Nissle 1917 CPS, named heparosan and classified as K5 antigen (https://www.iith.ac.in/EK3D/) has been shown to induce the secretion of IL-10 or IL-12 by Caco-2 cells, leading to alleviation of intestinal inflammation.^200,201^

*Bf. bifidum* strain PRI1, a potent inducer of Foxp3+ Treg cells, produces different cell surface polysaccharides, including charged PGβG and neutral CSGG (Table 7). CSGG (and not PGβG) facilitated the induction of Treg cells with the branched β-(1→6)-glucan being the proposed effector molecule. CSGG efficiently recapitulated the activity of whole bacteria and acted via regulatory dendritic cells through a partially Toll-like receptor 2-mediated mechanism. Treg cells induced by *Bf. bifidum* PRI1 or purified CSGG displayed stable and robust suppressive capacity in experimental colitis, highlighting the immunomodulatory potential of CSGG and CSGG-producing microbes.^179^

### 3.2. Biological roles of microbial EPS in the gut

EPS can be loosely attached to the bacterial cell surface or secreted.^202,203^ EPS have multiple functions: they can confer protection against desiccation, they contribute to the uptake of nutrients, they reduce the exposure to toxic compounds, and enable the bacteria to attach to surfaces through biofilm formation.^204^ In the gut, EPS have mainly been characterized from *Bifidobacterium* and *Lactobacillus* species. These are encoded by *eps* gene clusters encompassing genes encoding glycosyltransferases for the biosynthesis of the repeating units, proteins involved in the polymerization and export of these units, and other genes of unknown functions, including mobile elements.^205^ However, genes involved in the regulation of polysaccharide synthesis have only been identified in lactobacilli but not in bifidobacterial eps clusters (for a review see Castro-Bravo et al., 2018.^206^ The *Bifidobacterium* genus contains nine *eps* gene clusters conserved among different bifidobacterial species and a further 44 unique *eps* loci, accounting for a large part of the inter(sub)species variability across bifidobacterial genomes.^174,207^

EPS produced by strains from *Bifidobacterium* and *Lactobacillus* provide a protective surface layer interacting with the surrounding environment (for a review, see Castro-Bravo et al).^206^ and beneficial effects in the gut.^9^

A large panel of EPS has been isolated and characterized from the *Bifidobacterium* genus (Table 7). These have been shown to protect the bifidobacterial species against threats that can be found in the GI tract, therefore helping their persistence in the gut.^208^ In addition, bifidobacterial EPS have been implicated in the modulation of the gut microbiota.^209^ For example, *Bf. longum* subsp. *longum* YS108R EPS helped maintain the gut microbiota composition in the dextran sodium sulfate (DSS)-induced colitis mouse model.^210^ In contrast, *Bf. breve* strain IPLA20004 EPS has been shown to inhibit the growth of the commensal bacterium *B. thetaiotaomicron* strain DSM-2079 *in vitro*.^211^ Furthermore EPS from *Bifidobacteria* species play a crucial role in host immunomodulation.^9^ For example, *Bf. breve* strain UCC2003 EPS promoted the reduction in immune reactivity from the host through the reduction of B cell response, while it was also implicated in the reduction of gut pathogen *Citrobacter rodentium* colonization in mice.^212^ Also, *Bf. longum* strain BCRC 14,634 EPS induced the secretion of anti-inflammatory IL-10 and reduced LPS-induced pro-inflammatory TNF-α production in J774A.1 macrophages cells.^213^ In addition, *Bifidobacterium* EPSs have been shown to counteract the cytotoxic effect of some pathogen toxins (like those from *Bacillus cereus* and *Streptococcus pyogenes*) in epithelial Caco2 cells and rabbit erythrocytes *in vitro*.^214^ In a recent study, 21 strains belonging to *Bifidobacterium* and *Lactobacillus* species were isolated from healthy infant stool and screened for their EPS-producing ability. *Bf. longum* XZM1 showed highest EPS production; the purified 4023 Da EPS, composed of Man, Glc, Gal, Ara, and Fuc, exhibited high scavenging abilities toward hydroxyl than 1,1-diphenyl-2-picrylhydrazyl free radical.^215^

EPS from LAB have been largely studied for their biotechnological properties.^16,216^ However, more recent work focused on their role in promoting gut homeostasis through modulation of the gut microbiota and gut barrier function.^9^ For example, EPS isolated from *Lb. rhamnosus* strain ZFM231, when administered to DSS-induced colitis mice, was shown to restore gut microbiota diversity and composition at the phylum and genus level.^217^ *Lb. johnsonii* strain FI9785 produces two EPS types of different structures, a HoPS and a HePS, which have been implicated in gut colonization and competitive exclusion of the pathogen *Clostridium perfringens*.^218^ *Lcp. plantarum* T1 (formerly *Lactobacillus plantarum*), largely found in food-derived products, produces an EPS which can reduce the relative abundance of pathogenic bacteria such as *Escherichia-Shigella*, *Citrobacter*, *Fusobacterium*, *Parasutterella*, and *Lachnoclostridium* while increasing the level content of beneficial bacteria such as *Bacteroides*, *Blautia*, *Phascolarctobacterium*, *Bifidobacterium*, *Parabacteroides*, and *Subdoligranulum in vitro*.^219^ *Lactobacillus* EPS can also influence gut barrier function through the modulation of the expression or distribution of tight junction proteins in the intestinal epithelium, as shown for the EPS (HePS) from *Lcp. plantarum* strain HY7714, which has been reported to upregulate ZO-1 and occludin in Caco-2 cells.^220^

Finally, LAB EPS species play a crucial role in host immunomodulation.^9^ *Lb. reuteri* EPS has been implicated in immunotolerance, as shown, for example, for *Lb. reuteri* 100–23 EPS whose presence increased the proportion of regulatory T cells (Foxp3+) in mouse^221^ or with *Lb. reuteri* L26 EPS (HoPS), which led to reduced pro-inflammatory cytokine expression (such as TNF-α, IL-8, and IL-6) in IPEC-J2 cell line after *Salmonella* Typhimurium infection.^222^ EPS from *Lb. mucosae* CCFM1273 has been shown to alleviate disease symptoms and colonic pathological damage in DSS-induced colitis mice by improving gut barrier function and inhibiting epithelial cell apoptosis, increasing propionate in the gut, which inhibited the Fas/Fasl pathway, and by decreasing LPS level, which in turn inhibited the TLR4/NF-κB pathway.^223,224^ The EPS from *Leu. mesenteroides* NTM048 strain has been shown to induce total and antigen-specific IgA production by Peyer’s patch cells and modulate Th1 and Th2 cell-mediated responses in splenocytes *in vitro*.^225^ Oral administration of *Leu. mesenteroides* EPS in mice induced fecal IgA production, up-regulated retinoic acid synthase and growth factor-β receptor expression, and increased the CD3+ T-cell population and the ratio of CD4+ T-cells/CD8+ T-cells, thereby strengthening the mucosal barrier.^225^ EPS from *Lb. rhamnosus* KL37 strain showed production of both anti-inflammatory (IL-10) and pro-inflammatory (TNF-α, IL-6, IL-12) cytokines in mouse macrophages and TNF-α production could be reverted by pretreatment of macrophages with specific inhibitors of p38 and ERK MAPKs.^226^ These immunomodulation effects can also vary depending on the EPS fraction and molecular weight, even within the same species. For instance, purified EPS from *Lb. confusus* TISTR 1498 strain showed no immunomodulatory activity, while, when partially hydrolyzed, the lower molecular weight EPS induced the production of pro-inflammatory mediator nitric oxide and various cytokine production such as TNF-α, IL-1β, IL-6 and IL-10 in RAW264.7 cells through the activation of NF-κB and JNK pathways.^42^

Some EPS from LAB strains have been studied for their antitumor activities. For example, the EPS from *Lb. acidophilus* 10307 has been shown to induce cytotoxicity in cancer model cell lines like HCT15 and Caco2^227^; and EPS from *Lb. rhamnosus* ATCC 9595 has been shown to inhibit the growth of cancer cell lines PANC1 and HT-29,^228^ while *Lb. casei* SB27 EPS showed anti-proliferative effect in HT-29 cells.^229^ Administration of EPS from *Lb. plantarum* WLPL09 (EPS-09) in melanoma-bearing B16F10 mice showed antitumor activity by inducing apoptosis of tumor cells, inhibiting tumor angiogenesis, improving immunity, modulating the gut microbiota composition by increasing the abundance of bacteria in the *Ruminococcus* genus, and reducing the abundance of bacteria in *Prevotella*, *Akkermansia* and *Oscillospira* genera.^230^ EPS from LAB can also exhibit antioxidant activity, as recently reviewed.^41^

The EPSs produced by *S. thermophilus* strains have been implicated in the initial interactions with several phages of this species, as recently reviewed.^231^ In addition, an *vitro* analysis of phage-host interactions between eight EPS-producing *S. thermophilus* strains (CRL419, 638, 804, 810, 815, 817, 821, 1190) and five streptococcus-specific phages isolated from fermented foods revealed that the CPS layer surrounding the *S. thermophilus* cells could play a role in the adsorption of these specific phages to the cells.^232^

Together, these studies suggest that EPS could be an effective dietary supplement to counteract infection, inflammation, and tumorigenesis.

### 3.3. Biological roles of microbial CWPS in the gut

CPWS from *Enterococci*, *Lactococci* and *Streptococci* are characterized by RRP decorated with side chains of different sizes and carbohydrate composition, sometimes referred to as PSP.

The CPWS from the food commensal *Lcp. plantarum* IMB19 (LpIMB19), a rhamnose-rich heteropolysaccharide (RHP) with immunomodulatory properties,^96^ was found to contribute to LpIMB19 antitumor immunity in mouse tumor models.^233^ RHP was the major effector responsible for the recruitment and expansion of tumor-specific CD8+ T cells, and purified RHP could suppress murine melanoma growth in mice. *In vitro*, LpIMB19 CPS induced highly significant numbers of IFNγ+CD8+ T cells in a dose-dependent manner, while PG had a moderate impact and EPS had no effect.^233^ Bioinformatics analyses revealed no differences in CPS cluster gene copies between LpIMB19 and the closely related strain *Lcp. plantarum* KCTC21024, which displays no antitumor effect *in vitro* or in tumor models, which indicates that expression levels or the presence of additional CPS biosynthetic genes or modifications and structural variations in the polysaccharides of these strains could lead to the observed differences in phenotypes.^233^ These studies underscore the importance of in-depth structural characterization of the CPS produced by bacterial strains of the same species. In LAB, CWPS have also been reported to be the attaching point, or receptors, used by phages.^231,234^

Recently, CWPS have been identified in the Gram-positive human gut symbiont *R. gnavus*.^170^ *R. gnavus* is present from the early life of infants^235–237^ and persists across the lifetime as a component of the adult gut microbiota, where it is present in 90% of human faecal samples from healthy adults.^238–240^ *In vitro*, *R. gnavus* strains and their purified CWPS, glucorhamnan-I (characterized in *R. gnavus* ATCC 29149) and glucorhamnan-II (characterized in *R. gnavus* E1 and *R. gnavus* ATCC 35913) induced strain-specific production of cytokines and chemokines in bone-marrow derived dendritic cells (mBMDCs)^122,123^ and NF-κB activation in reporter cells.^122^ The synthesized pentasaccharide repeat of glucorhamnan-I was shown to be sufficient to induce an immune response and a release of inflammatory cytokines like TNF-α and IL-6 from mBMDCs through TLR4 recognition.^241^ Among the three strains tested (*R. gnavus* ATCC 29149, *R. gnavus* E1, and *R. gnavus* ATCC 35913), *R. gnavus* ATCC 35913 was found to be the most immunogenic strain, likely due to the absence of an additional capsular polysaccharide layer, while the purified glucorhamnans from the three strains showed activation of TLR4 reporter cells.^122^

Bioinformatics analyses revealed the presence of a glucorhamnan biosynthesis cluster which appeared conserved across all *R. gnavus* strains.^123^ This cluster contains 23 genes and variable numbers of glycosyltransferases, suggesting that the composition of residues within the glucorhamnan structure is different among *R. gnavus* strains. In addition to glucorhamnan, some strains of *R. gnavus* display on their cell surface a large molecular weight CPS ( >100 kDa). Monosaccharide analysis revealed that the capsule is composed of Glc, *N*-acetylquinovosamine, and GalNAc in a 6:2:1 stoichiometry, in accordance with the *cps* gene cluster of these strains, which contains nine glycosyltransferases, one for each of the monosaccharides in the repeating unit.^172^ This CPS has been shown to be tolerogenic as encapsulated *R. gnavus* strains induced little to no cytokine production by innate immune cells *in vitro* and *in vivo*.^172^ Gnotobiotic mice colonized with CPS^+^ *R. gnavus* strain RJX1120 had lower amount of T cells accumulating in the lamina propria with a higher percentage of FOXP3±Treg cells, while a higher percentage of CD62L^+^-T cells were induced in the lamina propria of gnotobiotic mice colonized with the CPS^−^ strain RJX1125.^172^ Also, *R. gnavus* RJX1120 CPS^+^ strain inhibited the production of IL-1β and TNF-α *in vivo*, as compared to *R. gnavus* RJX1125 CPS^−^ strain.^172^ These strain-specific differences in *R. gnavus* cell surface glycosylation and host response underscore the importance of studying bacteria-host interaction at the strain level.^238^

## 4. Conclusions and perspectives

All bacteria produce a rich variety of glycans, and the bacteria found in the human gut are no exception. The structural diversity of these glycans is underpinned by the type of building blocks, the nature of the monosaccharides, the way these are assembled, and their molecular weight, all influence their structural and biological functions. When part of the membrane, these glycans are referred to as CPS and CWPS, while glycans secreted in the medium are named EPS. While this review focused on CPS, EPS and CWPS, the complexity of the bacterial cell wall is further increased by the presence of other glycoconjugates such as teichoic acids which have been implicated in various functions, including cell wall maintenance and shape, cell division, gut colonization, resistance to antimicrobial agents and biofilm formation.^242^ Another example is the presence of glycosylated surface layer proteins (S-layer proteins) that can form crystalline arrays on the cell surface of many bacteria and archaea, providing structural stability and protection against environmental stresses, modulating immune responses, and enhancing mucosal homeostasis.^243^

Collectively, these diverse cell surface-associated polysaccharides and their interrelationship contribute to the dynamic interplay between gut commensal bacteria and the host environment, influencing various aspects of microbial ecology and host-microbe interaction. While gut bacteria produce polysaccharides for their own benefit to protect themselves from the host immune system, phages, or environmental challenges, *in vitro* and pre-clinical studies support the importance of cell surface glycans as major players in influencing host health.

The combined diversity of microbes within the gut microbiota and microbial polysaccharide structures provides a vast array of different glycans in the gut environment that could be harnessed as prebiotics or therapeutics to benefit human health.^244^ However, a major bottleneck in this field of research remains our capacity to study their detailed structure-function relationships. Currently, the literature is split into in-depth structural studies, which lack biological investigations of these molecules, or functional studies where, in many instances, the characterization of these molecules is limited to monosaccharide composition. This is symptomatic of a wider issue in the field of glycobiology where the capacity to conduct detailed structural characterization of glycans is limited due to the absence of accessible and high-throughput methods.

Indeed, the study of bacterial polysaccharides remains challenging since their structure is heterogeneous and chemically complex, as demonstrated by the rich variety of monosaccharides in nonpathogenic bacteria found in the gut. Unlike DNA and protein, the synthesis of glycans is not template-driven, so their structure cannot be predicted *a priori*, and relies on extensive wet chemistry to determine their composition and their glycosidic linkages. In addition, the monosaccharide sequence and their anomeric configuration can only be evaluated by NMR, whereby proton chemical shifts occur in a narrow range of values,^245^ and the study of the NMR spectra requires skilled manual annotation. New developments in mass spectrometry (MS) methods have pushed forward the study of small- or medium-sized glycans, glycolipids, and glycoproteins.^246^ However, the study of high molecular weight glycans still lies outside the boundaries of MS technical potential and often requires their depolymerization to be studied by these approaches.^246^ Improvements in glycan analytical techniques and bioinformatics will play an increasingly crucial role in addressing issues related to the structure of polysaccharides, and a better integration with the biological data is required to advance our understanding of the functional features influencing health and realize their full potential value in a variety of biotechnological and biomedical applications. While most studies investigating the biological effects of bacterial polysaccharides rely on intestinal cell lines, primary immune cells, or mouse models, the development of advanced *in vitro* human models, such as gut organoid-on-chip systems, will help provide novel insights on gut barrier function and immune response and accelerate translation of the findings to humans. Future work is needed to optimize the large-scale production, characterization, and formulation of EPS/CPS/CWPS that could be used as prebiotics or food additives as part of novel nutritional strategies promoting human health.

## Data Availability

All data used in this review are publicly available, hyperlinks to public databases are provided wherever relevant, and no additional source data are required.
